# New benzothiazole hybrids as potential VEGFR-2 inhibitors: design, synthesis, anticancer evaluation, and *in silico* study

**DOI:** 10.1080/14756366.2023.2166036

**Published:** 2023-01-24

**Authors:** Mohammad M. Al-Sanea, Abdelrahman Hamdi, Ahmed A. B. Mohamed, Hamed W. El-Shafey, Mahmoud Moustafa, Abdullah A. Elgazar, Wagdy M. Eldehna, Hidayat Ur Rahman, Della G. T. Parambi, Rehab M. Elbargisy, Samy Selim, Syed Nasir Abbas Bukhari, Omnia Magdy Hendawy, Samar S. Tawfik

**Affiliations:** a Department of Pharmaceutical Chemistry, College of Pharmacy, Jouf University, Sakaka, Saudi Arabia; b Department of Pharmaceutical Organic Chemistry, Faculty of Pharmacy, Mansoura University, Mansoura, Egypt; c Department of Medicinal Chemistry, Faculty of Pharmacy, Mansoura University, Mansoura, Egypt; d Department of Pharmacognosy, Faculty of Pharmacy, Mansoura University, Mansoura, Egypt; e Department of Pharmacognosy, Faculty of Pharmacy, Kafrelsheikh University, Kafrelsheikh, Egypt; f Department of Pharmaceutical Chemistry, Faculty of Pharmacy, Kafrelsheikh University, Kafrelsheikh, Egypt; g Department of Clinical Pharmacy, College of Pharmacy, Jouf University, Sakaka, Saudi Arabia; h Department of Pharmaceutics, College of Pharmacy, Jouf University, Sakaka, Saudi Arabia; i Department of Clinical Laboratory Sciences, College of Applied Medical Sciences, Jouf University, Sakaka, Saudi Arabia; j Department of Pharmacology, College of Pharmacy, Jouf University, Aljouf, Saudi Arabia

**Keywords:** 2-Aminobenzothiazole, thiazolidine-24-diones, cyanothiouracils, 134-thiadiazoles, VEGFR-2 inhibition

## Abstract

A new series of 2-aminobenzothiazole hybrids linked to thiazolidine-2,4-dione **4a–e**, 1,3,4-thiadiazole aryl urea **6a–d,** and cyanothiouracil moieties **8a–d** was synthesised. The in vitro antitumor effect of the new hybrids was assessed against three cancer cell lines, namely, HCT-116, HEPG-2, and MCF-7 using Sorafenib (SOR) as a standard drug. Among the tested compounds, **4a** was the most potent showing IC50 of 5.61, 7.92, and 3.84 µM, respectively. Furthermore, compounds **4e** and **8a** proved to have strong impact on breast cancer cell line with IC50 of 6.11 and 10.86 µM, respectively. The three compounds showed a good safety profile towards normal WI-38 cells. Flow cytometric analysis of the three compounds in MCF-7 cells revealed that compounds **4a** and **4c** inhibited cell population in the S phase, whereas **8a** inhibited the population in the G1/S phase. The most promising compounds were subjected to a VEGFR-2 inhibitory assay where **4a** emerged as the best active inhibitor of VEGFR-2 with IC50 91 nM, compared to 53 nM for SOR. In silico analysis showed that the three new hybrids succeeded to link to the active site like the co-crystallized inhibitor SOR.

## Introduction

Cancer is a complicated world spread fatal illness that affects several organs in the human body and represents a serious challenge to the health and welfare of humanity^1–3^. Rapid proliferation is the most common property shared among all types of cancer^4^. This proliferation involves highly complex and interconnected molecular pathways, this multifarious nature forces using multi-target cancer treatment techniques^5^. So, the discovery and development of new therapeutic agents with this anti-proliferative activity with selective cytotoxicity are needed urgently. Unfortunately, chemotherapeutic anticancer treatment causes several adverse effects, including multiple drug resistance, unwanted side effects, adverse events, and bad selectivity. Due to the previously‐mentioned shortcomings, there is tremendous need to discover and develop new, potent, safe, and efficacious therapeutic agents with anti-proliferative activity and selective cytotoxicity. The rapid expansion in the number of new anti-cancer drugs with versatile mechanisms of action has successfully emphasised traditionally used chemotherapeutic agents; these agents are used in combination with traditional agents. Although they acquire various mechanisms, most new drugs are thought to induce apoptosis of cancer cells or their supportive vascularity^6^.

**Figure 1.**
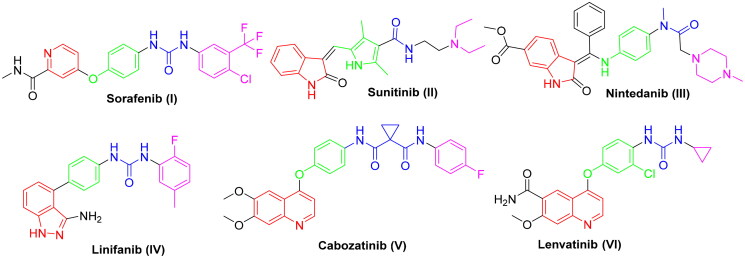
The necessary pharmacophoric properties of some FDA and clinically approved VEGFR-2 inhibitors.

Protein kinases (PKs) stand for cell function regulation^7^. Dysfunction of PKs leads to several diseases such as inflammation, metabolic diseases and ultimately cancer. Thus the utilisation of PK-inhibitors is regarded as a promising strategy to inhibit cancer progression^8^, especially in cancers known with mutations and alterations of PKs which contribute to development of drug resistance^9^. VEGFR-2 is a PK receptor located on blood vessels and regulates angiogenesis^10^. It is highly expressed in many types of cancer. Hence, it’s considered as a main target for many clinically approved anticancer agents (Figure 1)^11^.

SAR analysis of various VEGFR-2 inhibitors has revealed four common pharmacophoric features as demonstrated in Figure 1. These features include (i) hinge binder which is composed of a flat aromatic or heteroaromatic system containing one N at least and fills the catalytic ATP-binding pocket^12^. Moreover, X-ray structure analysis in different inhibitors linked to VEGFR-2 enzyme demonstrated that there is a sufficient space around the terminal heteroaromatic core available for various substituents^13^. (ii) A spacer group that occupies the linker region between the hinge region and the DFG-domain of the enzyme^14^. (iii) A pharmacophoric functional group consists of a moiety of hydrogen bond donor-acceptor pair (e.g. amide or urea) which binds with two essential amino acids (Glu885 and Asp1046) in the DFG (Asp-Phe-Gly) motif^15^. (iv) A hydrophobic tail (present only in type II inhibitors) which occupies the back allosteric site created when phenylalanine residue of the DFG loop flips out of its lipophilic pocket making the DFG motif adopting the inactive conformation. So, various hydrophobic bindings mostly occur in this allosteric binding site^16^.

Generally, due to the nature of VEGFR-2 enzyme binding site, its inhibitors display wide diversity in structure such as picolinamide (e.g. Sorafenib) SOR (**I**), indole (e.g. Sunitinb **II** and Nintedanib **III**), indazole (e.g. Linifanib **IV**), and quinolone (e.g. Cabozatinib **V** and Lenvatinib **VI**) (Figure 1)^17–23^.

Benzothiazole is a privileged chemical scaffold possessing a wide spectrum of pharmacological effects including, anti-diabetic^24^, fungicidal^25^, anti-microbial^26^, analgesic^27^, CNS depressant^28^, as well as anticancer activity^29–32^. This moiety revealed outstanding and prevalent pharmacological and biological effect against various kinds of cancer cell lines as HeLa, SW480, HepG2, mammary and ovarian tumour cell lines, colon, non-small-cell lung and breast subpanels cell lines, and HCC^33–36^.

The benzothiazole and its isosteres like the indole ring have proved to exhibit promising antitumor activity. The structure–activity relationship for the various derivatives revealed an excellent understanding of the behaviour of benzothiazole moiety in the field of cancer therapy against different cancer cell line. This moiety has also been explored for its therapeutic potential. Benzothiazole‐based derivatives have emerged as effective enzyme inhibitors against EGFR, VEGFR, PI3K, topoisomerases, and thymidylate kinases. Some of these inhibitors have entered different phases of clinical trials. In addition, aryl benzothiazole, aminobenzothiazole and other structural benzothiazole hybrids have attracted outstanding attention in the search for new chemotherapeutic agents as they exhibited effective cytotoxic activity in *in vivo* and *in vitro* models^37–42^.

**Figure 2.**
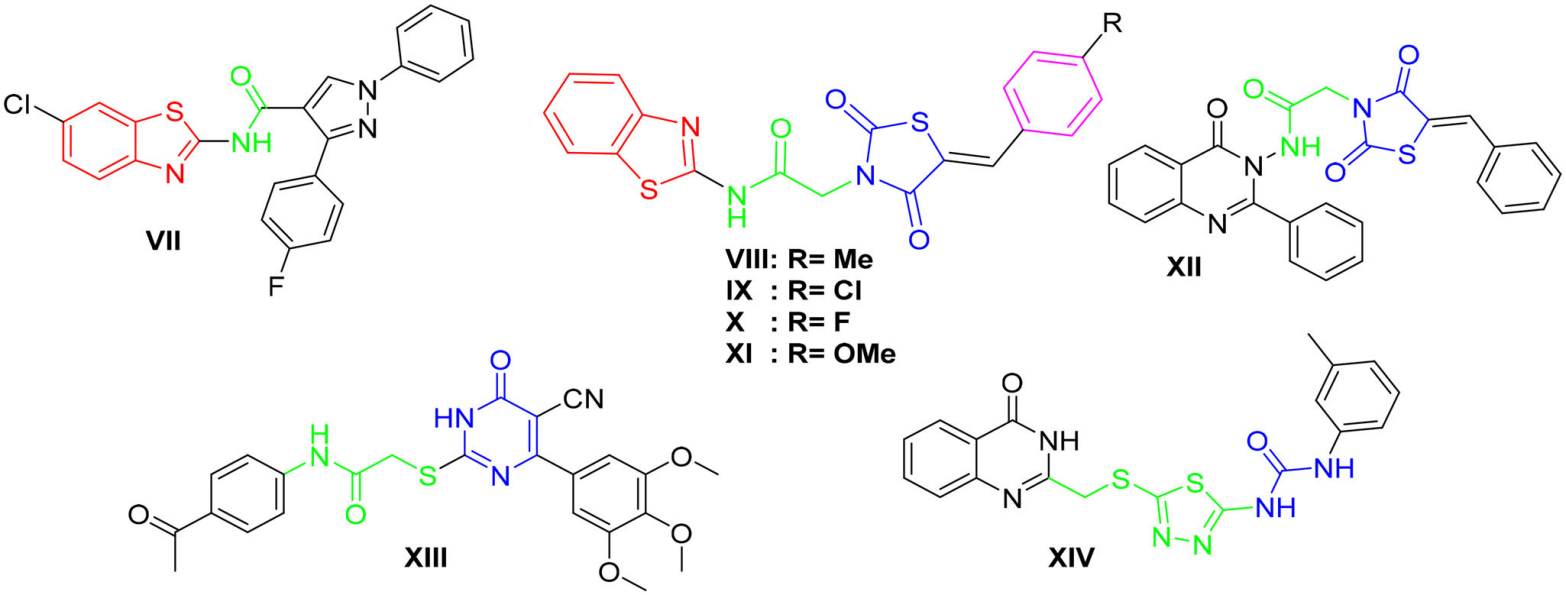
Representative examples of some reported anticancer VEGFR-2 inhibitors carrying 2-aminobenzothiazole scaffold, thiazolidine-2,4-dione, cyanothiouracil, and thiadiazole-urea pharmacophores.

Furthermore, during the last years, there have been several attempts to develop different benzothiazole surrogates with unique and promising profile as antitumor agents through inhibition of protein kinases^43–46^. In this context, 2-aminobenzothiazole derivatives were found to be very effective scaffolds as will be shown in this work^47^. Recently, V.G. Reddy et al. proposed a series of 2-aminobenzothiazole-pyrazoles as a new group of potent VEGFR-2 inhibitors with outstanding anticancer effect in the low micromolar range towards several cancer cell lines. Within this series, compound **VII** exerted the highest activity with a VEGFR-2 IC_50_ of 97 nM (Figure 2)^48^. Furthermore, K. El-Adl et al. disclosed the 2-aminobenzothiazole-thiazolidinedione hybrids **VIII–XI** as new VEGFR-2 inhibitors with IC_50_ 150–210 nM, introducing them as a novel start to produce promising inhibitors against VEGFR-2 (Figure 2)^45^.

In addition, different scaffolds such as thiazolidine-2,4-dione (e.g. compound **XII**)^14^, 6-aryl-5-cyanothiouracil (e.g. compound **XIII**)^49^, and thiadiazole-urea (e.g. compound **XIV**)^50^ were reported as anticancer activity enhancers with potent VEGFR-2 inhibitory activity (Figure 2).

Counting on the ligand-based design of drugs, molecular hybridisation approach is concerned with the combination of two or more bioactive groups to produce hybrids showing enhanced bioactivity, particularly, when applied in the search for new effective antitumor candidates^51^. So that, the anticancer and VEGFR-2 inhibitory activity of benzothiazole are combined with anticancer activity enhancer in a new structure that is expected to exert more potent anticancer candidates that possess the same vital pharmacophoric characteristics of the old VEGFR-2 inhibitors (e.g. SOR) with bioisosteric alterations at four various sites.

So, in this work we depended on the following strategy to design novel VEGFR inhibitors. Firstly, we adopted a bioisosteric replacement of the pyridine ring of SOR with benzothiazole scaffold. In the second modification, various linkers with variable lengths were designed to occupy the spacer region. The linker may involve three or four atom bridges (acetamide and 2-sulfanylacetamide). In some instances, it is four atoms in addition to 1,3,4-thiadiazole moiety. These linkers were considered to take the place of the central aryl moiety of the lead structure to enhance the flexibility which consequently should improve the affinity of VEGFR-2 binding. The main modifications focussed on the region representing the HBA/HBD pharmacophore where moieties with effective anticancer potency such as thiazolidine-2,4-dione, cyanothiouracil, or urea were used instead of the pharmacophore of unsymmetrical diaryl urea scaffolds of SOR.

**Figure 3.**
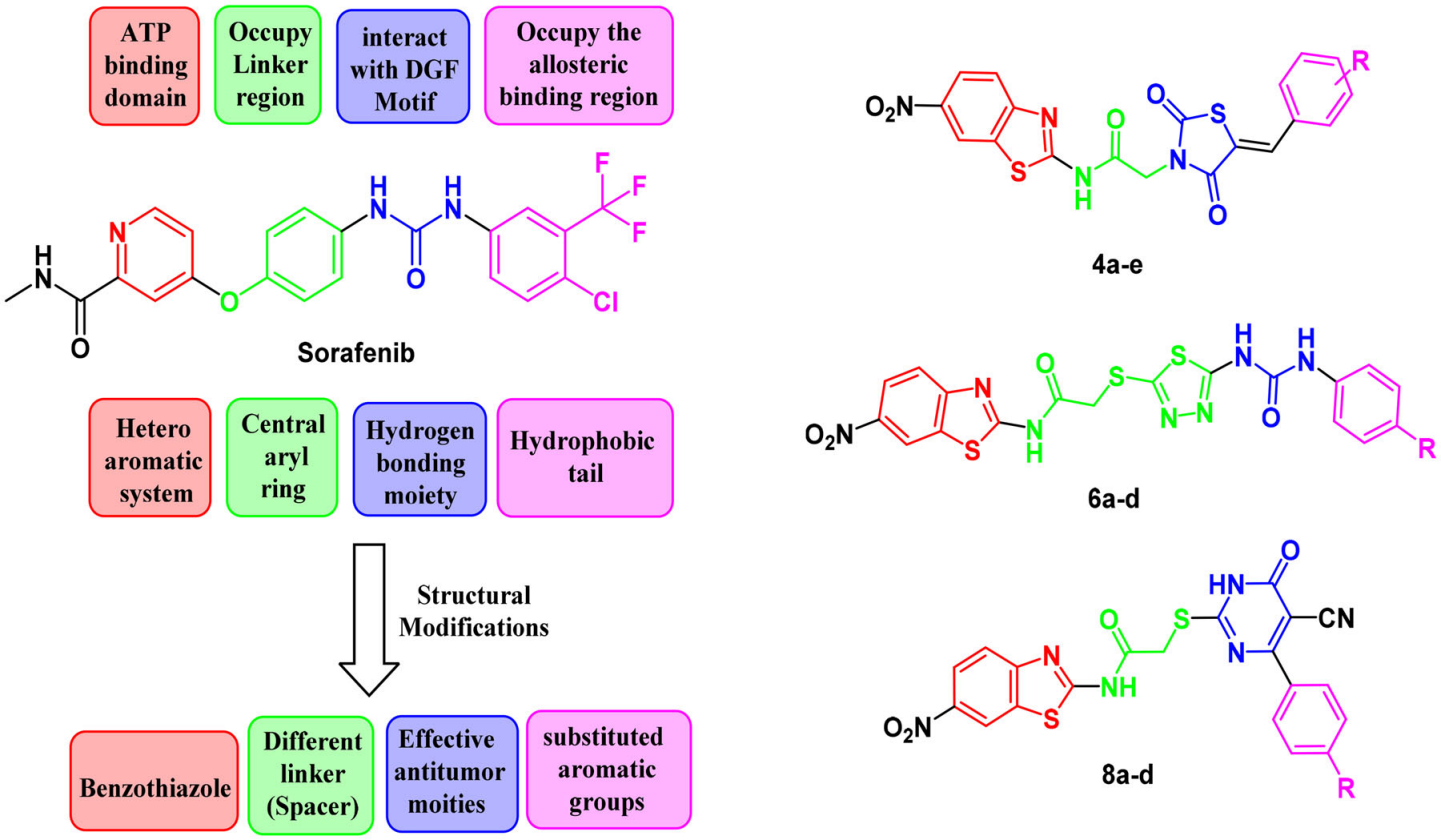
The fundamental structural requirements for SOR and rational of design of the novel postulated VEGFR-2 inhibitors.

Then, the aromatic terminal of the lead structure was replaced by another different hydrophobic system attached to either electron withdrawing or electron donating group that may affect the activity of the new hybrids allowing studying SAR of such hybrids as potential anti-tumour candidates with potential inhibitory potencies against VEGFR-2. All alteration pathways and design rationale were illustrated in Figure 3.

## Material and methods

### Chemistry

The mp (°C) were recorded using Stuart melting point apparatus (SMP 30) and are uncorrected. IR (KBr) was measured on FT-IR 200 spectrophotometer (ύ cm^−1^), Faculty of Pharmacy, Mansoura University. ^1^H-NMR and ^13^C-NMR were performed in (DMSO-*d_6_*) at ^1^HNMR (400 MHz), ^13^CNMR (100 MHz) on an NMR spectrometer (*δ* ppm) using TMS as an internal standard, NMR Unit, Faculty of Pharmacy, Mansoura University. Abbreviations are as indicated: (s), singlet; (d), doublet; (t), triplet; (m), multiplet; (br), broad. Mass spectra were performed in Thermo Scientific GC/MS model ISQ at the Regional Centre for Mycology and Biotechnology (RCMB), Al-Azhar University, Egypt. Microanalyses were carried out at Cairo University on PerkinElmer 240 elemental analyser for elements C, H, and N, results were obtained within the accepted limits. Compounds were visualised with 254 nm UV lamp. Chemicals and reagents were obtained from Aldrich Chemicals Co, USA, and commercial sources. The key precursor compound **2** and intermediate compounds **3a–e**, **5a–d**, and **7a–d** were synthesised following reported procedures in literature^50^^,^^52–54^.

#### General method for the preparation of thiazolidine-2,4-dione derivatives (4a–e)

To a solution of the appropriate salt derivative **3a–e** (0.3 mmol) in DMF, the chloride derivative **2** (0.3 mmol) was added. Then, the mixture was refluxed all night. After reaction was complete (TLC), the mixture was poured into crushed ice; the precipitate obtained was filtered off and recrystallized from ethanol.

#### 2-(5-(4-Fluorobenzylidene)-2,4-dioxothiazolidin-3-yl)-N-(6-nitrobenzo[d]thiazol-2-yl)acetamide (4a)

Brown solid; (0.08 g, 58%). M.p. 199–201 °C. IR (*ν*max/cm^−1^): 3445 (NH), 1740, 1680 (C = O). ^1^H NMR (400 MHz, DMSO-*d_6_*) δ 13.30 (s, 1H, NH), 9.10 (s, 1H, thiazole-H), 8.30 (d, *J* = 7.7 Hz, 1H, thiazole-H), 8.1 (s, 1H, benzylidene-H), 7.9 (d, *J* = 7.7 Hz, 1H, thiazole-H), 7.73 (d, *J* = 8.5 Hz, 2H, phenyl-2H), 7.44 (d, *J* = 8.5 Hz, 2H, phenyl-2H), 4.76 (s, 2H, CH_2_). ^19^F NMR (377 MHz, DMSO-*d_6_*) δ −108.91 (s). ^13^C NMR (101 MHz, DMSO-*d_6_*) δ 167.5, 166.9, 166.8, 165.6, 164.9, 164.0, 143.7, 133.4, 133.2, 132.9, 132.7, 122.4, 121.0, 119.7, 117.3, 117.0, 44.3. MS m/z (%):458.37 (M^+^, 29.33). Anal. Calcd for C_19_H_11_FN_4_O_5_S_2_ (458.44): C, 49.78; H, 2.42; N, 12.22. Found: C, 49.55; H, 2.48; N, 12.12%.

#### 2-(5-(4-Methoxybenzylidene)-2,4-dioxothiazolidin-3-yl)-N-(6-nitrobenzo[d]thiazol-2-yl)acetamide (4b)

Orange solid; (0.092 g, 65%). M.p. 259–261 °C. IR (*ν*max/cm^−1^): 3451 (NH), 1739, 1683 (C = O). ^1^H NMR (400 MHz, DMSO-*d_6_*) δ 13.24 (s, 1H, NH), 9.08 (s, 1H, thiazole-H), 8.30 (d, *J* = 7.7 Hz, 1H, thiazole-H), 8.0 (s, 1H, benzylidene-H), 7.91 (d, *J* = 7.7 Hz, 1H, thiazole-H), 7.65 (d, *J* = 7.1 Hz, 2H, phenyl-2H), 7.15 (d, *J* = 7.1 Hz, 2H, phenyl-2H), 4.40 (s, 2H, CH_2_), 3.85 (s, 3H, OCH_3_). ^13^C NMR (101 MHz, DMSO-*d_6_*) δ 167.7, 167.0, 165.7, 163.6, 162.8, 161.9, 143.6, 134.4, 132.9, 132.7, 132.5, 122.3, 121.2, 119.7, 117.9, 115.5, 56.0, 44.1. MS m/z (%):470.59 (M^+^, 24.65). Anal. Calcd for C_20_H_14_N_4_O_6_S_2_ (470.48): C, 51.06; H, 3.00; N, 11.91. Found: C, 51.15; H, 3.08; N, 11.88.

#### 2-(5-(4-Bromobenzylidene)-2,4-dioxothiazolidin-3-yl)-N-(6-nitrobenzo[d]thiazol-2-yl)acetamide (4c)

Yellow solid; (0.099 g, 63%). M.p. 252–254 °C. IR (*ν*max/cm^−1^): 3450 (NH), 1747, 1691 (C = O). ^1^H NMR (400 MHz, DMSO-*d_6_*) δ 13.30 (s, 1H, NH), 9.09 (s, 1H, thiazole-H), 8.31 (d, *J* = 7.7 Hz, 1H, thiazole-H), 8.0 (s, 1H, benzylidene-H), 7.90 (d, *J* = 7.7 Hz, 1H, thiazole-H), 7.77 (d, *J* = 8.5 Hz, 2H, phenyl-2H), 7.63 (d, *J* = 8.5 Hz, 2H, phenyl-2H), 4.40 (s, 2H, CH_2_). ^13^C NMR (101 MHz, DMSO-*d_6_*) δ 172.6, 167.3, 166.9, 165.5, 163.5, 162.8, 143.7, 133.2, 132.9, 132.5, 132.2, 125.0, 122.4, 122.1, 121.3, 119.7, 44.3. MS m/z (%):519.53 (M^+^, 20.71). Anal. Calcd for C_19_H_11_BrN_4_O_5_S_2_ (519.35): C, 43.94; H, 2.13; N, 10.79. Found: C, 43.90; H, 2.14; N, 10.88%.

#### 2-(5-(4-Methylbenzylidene)-2,4-dioxothiazolidin-3-yl)-N-(6-nitrobenzo[d]thiazol-2-yl)acetamide (4d)

Beige solid; (0.076 g, 56%). M.p. 201–203 °C. IR (*ν*max/cm^−1^): 3430 (NH), 1740, 1688 (C = O). ^1^H NMR (400 MHz, DMSO-*d_6_*) δ 13.13 (s, 1H, NH), 9.09 (s, 1H, thiazole-H), 8.30 (d, *J* = 7.7 Hz, 1H, thiazole-H), 8.0 (s, 1H, benzylidene-H), 7.95 (d, *J* = 7.7 Hz, 1H, thiazole-H), 7.58 (d, *J* = 8.1 Hz, 2H, phenyl-2H), 7.40 (d, *J* = 8.1 Hz, 2H, phenyl-2H), 4.75 (s, 2H, CH_2_), 2.39 (s, 3H, CH_3_). ^13^C NMR (101 MHz, DMSO-*d_6_*) δ 167.6, 167.0, 165.7, 163.6, 162.8, 143.6, 134.5, 132.7, 130.8, 130.6, 130.5, 130.4, 122.3, 121.3, 120.2, 119.7, 44.2, 21.6. MS m/z (%):454.14 (M^+^, 19.79). Anal. Calcd for C_20_H_14_N_4_O_5_S_2_ (454.48): C, 52.85; H, 3.10; N, 12.33. Found: C, 52.70; H, 3.15; N, 12.32%.

#### 2-(2,4-Dioxo-5–(3,4,5-trimethoxybenzylidene)thiazolidin-3-yl)-N-(6-nitrobenzo[d]thiazol-2-yl)acetamide (4e)

Buff solid; (0.110 g, 69%). M.p. 149–151 °C. IR (*ν*max/cm^−1^): 3450 (NH), 1745, 1670 (C = O). ^1^H NMR (400 MHz, DMSO-*d_6_*) δ 13.30 (s, 1H, NH), 9.10 (s, 1H, thiazole-H), 8.32 (d, *J* = 7.7 Hz, 1H, thiazole-H), 8.0 (s, 1H, benzylidene-H), 7.93 (d, *J* = 7.7 Hz, 1H, thiazole-H), 7.01 (s, 2H, phenyl-2H), 4.76 (s, 2H, CH_2_), 3.86 (s, 6H, 2OCH_3_), 3.76 (s, 3H, OCH_3_). ^13^C NMR (101 MHz, DMSO-*d_6_*) δ 167.6, 166.9, 165.6, 163.4, 162.8, 153.8, 143.7, 140.2, 134.6, 132.7, 128.8, 122.4, 121.4, 120.3, 119.8, 108.2, 60.7, 56.5, 44.1. MS m/z (%):530.70 (M^+^, 31.02). Anal. Calcd for C_22_H_18_N_4_O_8_S_2_ (530.53): C, 49.81; H, 3.42; N, 10.56. Found: C, 49.74; H, 3.42; N, 10.51%.

### General method for the preparation of 1,3,4-thiadiazole derivatives (6a–d)

The appropriate thiol derivative **5a–d** (0.3 mmole) with K_2_CO_3_ (0.45 mmole) in acetone were left for stirring at rt for 30 min. Then, an equivalent amount of the chloride derivative **2** was added. The resulting mixture was refluxed overnight. Then it was poured into crushed ice; the formed solid was obtained and recrystallized from ethanol to afford the required hybrids.

#### 2-((5-(3-(4-Chlorophenyl)ureido)-1,3,4-thiadiazol-2-yl)thio)-N-(6-nitrobenzo[d]thiazol-2-yl)acetamide (6a)

Brown solid; (0.098 g, 62%). M.p. 241–243 °C. IR (*ν*max/cm^−1^): 3270 (NH), 1685 (C = O). ^1^H NMR (400 MHz, DMSO-*d_6_*) δ 12.91 (s, 1H, NH), 11.8 (br s, 1H, NH), 9.51 (s, 1H, NH), 9.08 (s, 1H, thiazole-H), 8.30 (d, *J* = 8.8 Hz, 1H, thiazole-H), 7.94 (d, *J* = 8.8 Hz, 1H, thiazole-H), 7.54 (d, *J* = 8.5 Hz, 2H, phenyl-2H), 7.35 (d, *J* = 8.5 Hz, 2H, phenyl-2H), 4.38 (s, 2H, CH_2_). ^13^C NMR (101 MHz, DMSO-*d_6_*) δ 168.4, 163.9, 157.9, 153.9, 143.5, 138.0, 133.1, 132.7, 129.7, 129.2, 127.0, 122.3, 121.2, 120.9, 119.6, 37.4. MS m/z (%): 521.08 (M^+^, 60.75). Anal. Calcd for C_18_H_12_ClN_7_O_4_S_3_ (521.98): C, 41.42; H, 2.32; N, 18.78. Found: C, 41.46; H, 2.12; N, 18.74%.

#### 2-((5-(3-(4-Methoxyphenyl)ureido)-1,3,4-thiadiazol-2-yl)thio)-N-(6-nitrobenzo[d]thiazol-2-yl)acetamide (6 b)

Yellow solid; (0.093 g, 60%). M.p. 269–271 °C. IR (*ν*max/cm^−1^): 3290 (NH), 1682 (C = O). ^1^H NMR (400 MHz, DMSO-*d_6_*) δ 13.11 (s, 1H, NH), 11.4 (br s, 1H, NH), 9.07 (br s, 2H, NH + thiazole-H), 8.30 (d, *J* = 8.0 Hz, 1H, thiazole-H), 7.93 (d, *J* = 8.0 Hz, 1H, thiazole-H), 7.38 (d, *J* = 7.1 Hz, 2H, phenyl-2H), 6.89 (d, *J* = 7.1 Hz, 2H, phenyl-2H), 4.37 (s, 2H, CH_2_), 3.73 (s, 3H, OCH_3_). ^13^C NMR (101 MHz, DMSO-*d_6_*) δ 167.4, 166.9, 163.2, 161.5, 158.9, 153.5, 143.8, 142.1, 132.5, 122.3, 119.9, 119.7, 119.6, 119.4, 114.3, 37.6, 56.1. MS m/z (%):517.52 (M^+^, 25.81). Anal. Calcd for C_19_H_15_N_7_O_5_S_3_ (517.56): C, 44.09; H, 2.92; N, 18.94. Found: C, 44.11; H, 2.91; N, 18.94%.

#### N-(6-Nitrobenzo[d]thiazol-2-yl)-2-((5–(3-(p-tolyl)ureido)-1,3,4-thiadiazol-2-yl)thio)acetamide (6c)

Brown solid; (0.085 g, 56%). M.p. 238–240 °C. IR (*ν*max/cm^−1^): 3283, 3173 (NH), 1683 (C = O). ^1^H NMR (400 MHz, DMSO-*d_6_*) δ 13.13 (s, 1H, NH), 11.02 (s, 1H, NH), 9.09 (s, 1H, NH), 8.97 (s, 1H, thiazole-H), 8.30 (d, *J* = 8.9 Hz, 1H, thiazole-H), 7.94 (d, *J* = 8.9 Hz, 1H, thiazole-H), 7.35 (d, *J* = 8.1 Hz, 2H, phenyl-2H), 7.13 (d, *J* = 8.1 Hz, 2H, phenyl-2H), 4.38 (s, 2H, CH_2_), 2.26 (s, 3H, CH_3_). ^13^C NMR (101 MHz, DMSO-*d_6_*) δ 168.4, 166.4, 163.8, 161.5, 153.9, 143.6, 142.4, 136.1, 132.7, 129.8, 122.3, 121.3, 119.7, 119.6, 119.4, 37.4, 20.9. MS m/z (%):501.66 (M^+^, 23.56). Anal. Calcd for C_19_H_15_N_7_O_4_S_3_ (501.56): C, 45.50; H, 3.01; N, 19.55. Found: C, 45.55; H, 2.99; N, 19.59%.

#### N-(6-Nitrobenzo[d]thiazol-2-yl)-2-((5–(3-phenylureido)-1,3,4-thiadiazol-2-yl)thio)acetamide (6d)

Beige solid; (0.094 g, 64%). M.p. 282–284 °C. IR (*ν*max/cm^−1^): 3290 (NH), 1683 (C = O). ^1^H NMR (400 MHz, DMSO-*d_6_*) δ 13.13 (s, 1H, NH), 11.03 (s, 1H, NH), 9.08 (br s, 2H, NH + thiazole-H), 8.30 (d, *J* = 8.0 Hz, 1H, thiazole-H), 7.94 (d, *J* = 8.0 Hz, 1H, thiazole-H), 7.47 (d, *J* = 6.6 Hz, 2H, phenyl-2H), 7.33 (d, *J* = 6.6 Hz, 2H, phenyl-2H), 7.11– 7.05 (m, 1H, phenyl-1H), 4.39 (s, 2H, CH_2_). ^13^C NMR (101 MHz, DMSO-*d_6_*) δ 168.4, 164.3, 163.8, 161.2, 153.9, 143.6, 138.9, 132.7, 129.4, 124.6, 123.7, 122.3, 121.3, 119.6, 119.4, 37.4. MS m/z (%):487.43 (M^+^, 28.83). Anal. Calcd for C_18_H_13_N_7_O_4_S_3_ (487.54): C, 44.34; H, 2.69; N, 20.11. Found: C, 44.24; H, 2.60; N, 20.31%.

### General method for the preparation of cyanothiouracil derivatives (8a–d)

The appropriate thiol derivative **7a–d** (0.3 mmole) with K_2_CO_3_ (0.45 mmole) in acetone were stirred at rt for half an hour. An equivalent amount of the chloride **2** was then added. The obtained mixture was refluxed overnight. The precipitate was filtered, washed with acetone to furnish the target compounds.

#### 2-((4-(4-Chlorophenyl)-5-cyano-6-oxo-1,6-dihydropyrimidin-2-yl)thio)-N-(6-nitrobenzo[d] thiazol-2-yl)acetamide (8a)

Beige solid; (0.082 g, 55%). M.p. 252–254 °C. IR (*ν*max/cm^−1^): 3496, 3155 (NH), 2219 (CN), 1667 (C = O). ^1^H NMR (400 MHz, DMSO-*d_6_*) δ 13.11 (s, 1H, NH), 9.05 (s, 1H, thiazole-H), 8.33 (d, *J* = 8.9 Hz, 1H, thiazole-H), 7.95 (d, *J* = 8.9 Hz, 1H, thiazole-H), 7.76 (d, *J* = 8.3 Hz, 2H, phenyl-2H), 7.26 (d, *J* = 8.3 Hz, 2H, phenyl-2H), 4.34 (s, 2H, CH_2_), 1.2 (s, 1H). ^13^C NMR (101 MHz, DMSO-*d_6_*) δ 168.2, 166.5, 166.4, 163.8, 162.1, 153.9, 143.6, 136.6, 134.4, 132.6, 130.7, 128.8, 122.4, 121.2, 119.6, 116.3, 93.8, 35.1. MS m/z (%):498.99 (M^+^, 23.03). Anal. Calcd for C_20_H_11_ClN_6_O_4_S_2_ (498.92): C, 48.15; H, 2.22; N, 16.84. Found: C, 48.28; H, 2.20; N, 16.77%.

#### 2-((4-(4-Bromophenyl)-5-cyano-6-oxo-1,6-dihydropyrimidin-2-yl)thio)-N-(6-nitrobenzo[d] thiazol-2-yl)acetamide (8b)

White solid; (0.114 g, 70%). M.p. 229–231 °C. IR (*ν*max/cm^−1^): 3450 (NH), 2217 (CN), 1668 (C = O). ^1^H NMR (400 MHz, DMSO-*d_6_*) δ 13.11 (s, 1H, NH), 9.04 (s, 1H, thiazole-H), 8.32 (d, *J* = 8.9 Hz, 1H, thiazole-H), 7.96 (d, *J* = 8.9 Hz, 1H, thiazole-H), 7.68 (d, *J* = 8.1 Hz, 2H, phenyl-2H), 7.38 (d, *J* = 8.1 Hz, 2H, phenyl-2H), 4.34 (s, 2H, CH_2_), 1.2 (s, 1H). ^13^C NMR (101 MHz, DMSO-*d_6_*) δ 168.2, 166.6, 166.3, 163.7, 161.9, 153.9, 143.6, 134.7, 132.6, 131.7, 130.9, 125.6, 122.4, 121.2, 119.6, 116.2, 93.9, 35.1. MS m/z (%):543.57 (M^+^, 19.70). Anal. Calcd for C_20_H_11_BrN_6_O_4_S_2_ (543.37): C, 44.21; H, 2.04; N, 15.47. Found: C, 44.02; H, 2.08; N, 15.55%.

#### 2-((5-Cyano-4-(4-methoxyphenyl)-6-oxo-1,6-dihydropyrimidin-2-yl)thio)-N-(6-nitrobenzo[d] thiazol-2-yl)acetamide (8c)

Off white solid; (0.105 g, 71%). M.p. 273–275 °C. IR (*ν*max/cm^−1^): 3451(NH), 2221 (CN), 1700, 1659 (C = O). ^1^H NMR (400 MHz, DMSO-*d_6_*) δ 13.17 (s, 1H, NH), 9.07 (s, 1H, thiazole-H), 8.32 (d, *J* = 8.2 Hz, 1H, thiazole-H), 7.97 (d, *J* = 8.2 Hz, 1H, thiazole-H), 7.81 (d, *J* = 8.4 Hz, 2H, phenyl-2H), 6.71 (d, *J* = 8.4 Hz, 2H, phenyl-2H), 4.37 (s, 2H, CH_2_), 3.61 (s, 3H, OCH_3_), 1.2 (s, 1H). ^13^C NMR (101 MHz, DMSO-*d_6_*) δ 168.5, 167.1, 165.2, 163.9, 160.5, 159.8, 153.9, 143.5, 132.9, 132.5, 130.1, 122.3, 121.1, 119.6, 116.9, 114.6, 93.1, 35.1, 55.8. MS m/z (%):494.61 (M^+^, 25.45). Anal. Calcd for C_21_H_14_N_6_O_5_S_2_ (494.50): C, 51.01; H, 2.85; N, 16.99. Found: C, 51.12; H, 2.80; N, 16.93%.

#### 2-((5-Cyano-6-oxo-4-(p-tolyl)-1,6-dihydropyrimidin-2-yl)thio)-N-(6-nitrobenzo[d]thiazol-2-yl)acetamide (8d)

Beige solid; (0.090 g, 63%). M.p. 210–212 °C. IR (*ν*max/cm^−1^): 3486, 3182 (NH), 2227 (CN), 1684 (C = O). ^1^H NMR (400 MHz, DMSO-*d_6_*) δ 13.13 (s, 1H, NH), 9.06 (s, 1H, thiazole-H), 8.33 (d, *J* = 8.9 Hz, 1H, thiazole-H), 7.97 (d, *J* = 8.9 Hz, 1H, thiazole-H), 7.66 (d, *J* = 8.0 Hz, 2H, phenyl-2H), 6.98 (d, *J* = 8.0 Hz, 2H, phenyl-2H), 4.36 (s, 2H, CH_2_), 2.12 (s, 3H, CH_3_), 1.2 (s, 1H). ^13^C NMR (101 MHz, DMSO-*d_6_*) δ 168.1, 167.6, 165.7, 163.8, 161.9, 153.97, 143.5, 142.1, 132.8, 132.7, 129.3, 128.9, 122.3, 121.1, 119.6, 116.4, 93.1, 35.1, 21.3. MS m/z (%):478.79 (M^+^, 19.92). Anal. Calcd for C_21_H_14_N_6_O_4_S_2_ (478.50): C, 52.71; H, 2.95; N, 17.56; S, 13.40. Found: C, 52.71; H, 2.92; N, 17.65; S, 13.47%.

### Biological evaluation

#### Antitumor screening

The *in vitro* anticancer activity of the new benzothiazole hybrids was evaluated by performing MTT assay as in the literature^51^^,^^55^^,^^56^.

#### In vitro VEGFR-2 kinase inhibitory assay

VEGFR-2 enzyme assay was performed as reported^57^.

#### Flow cytometry analysis of the cell cycle distribution

It was carried out using MCF-7 cell lines stained with PI and analysed by FACS Calibur flow cytometer as reported^51^^,^^58^.

#### Analysis of cellular apoptosis

The extent of apoptosis was evaluated on MCF-7 cells and Annexin V-FITC/PI apoptosis detection kit following the reported method^51^^,^^59^.

### In silico evaluation and ADME prediction

#### Molecular docking

Since compounds **4a**, **4e,** and **8a** showed significant inhibition to VEGFR-2, Molecular docking was utilised for shedding the light on the mode of binding of these compounds. Briefly, chemical structure of hybrids of interest was sketched by Chemoffice software^60^, converted to SDF file, then their 3 D structure was generated by MOE software and the file was saved as mol2 file^61^. The 3 D structure of (VEGFR-2) was obtained from PDB using the code (4ASD) and subjected to protein preparation protocol in MOE using default options.

The prepared PDB was exported to Leadit software^62^^,^^63^ and the active site was set as sphere with radius 6.5 Å around the co-crystallized ligand. The software was validated as previously reported^64^ and the RMSD was found to be 0.8. The 3 D structure of the compounds was selected as a library and was docked to the active site. Finally, the docked poses were inspected using Discovery studio visualiser to study their interaction with the binding site^65^.

#### In silico prediction of physicochemical properties and pharmacokinetic profile

For ADMET profiling, the free access of website (https://preadmet.bmdrc.kr/)^66^ was used for estimation.

## Results and discussion

### Chemistry

**Scheme 1.**
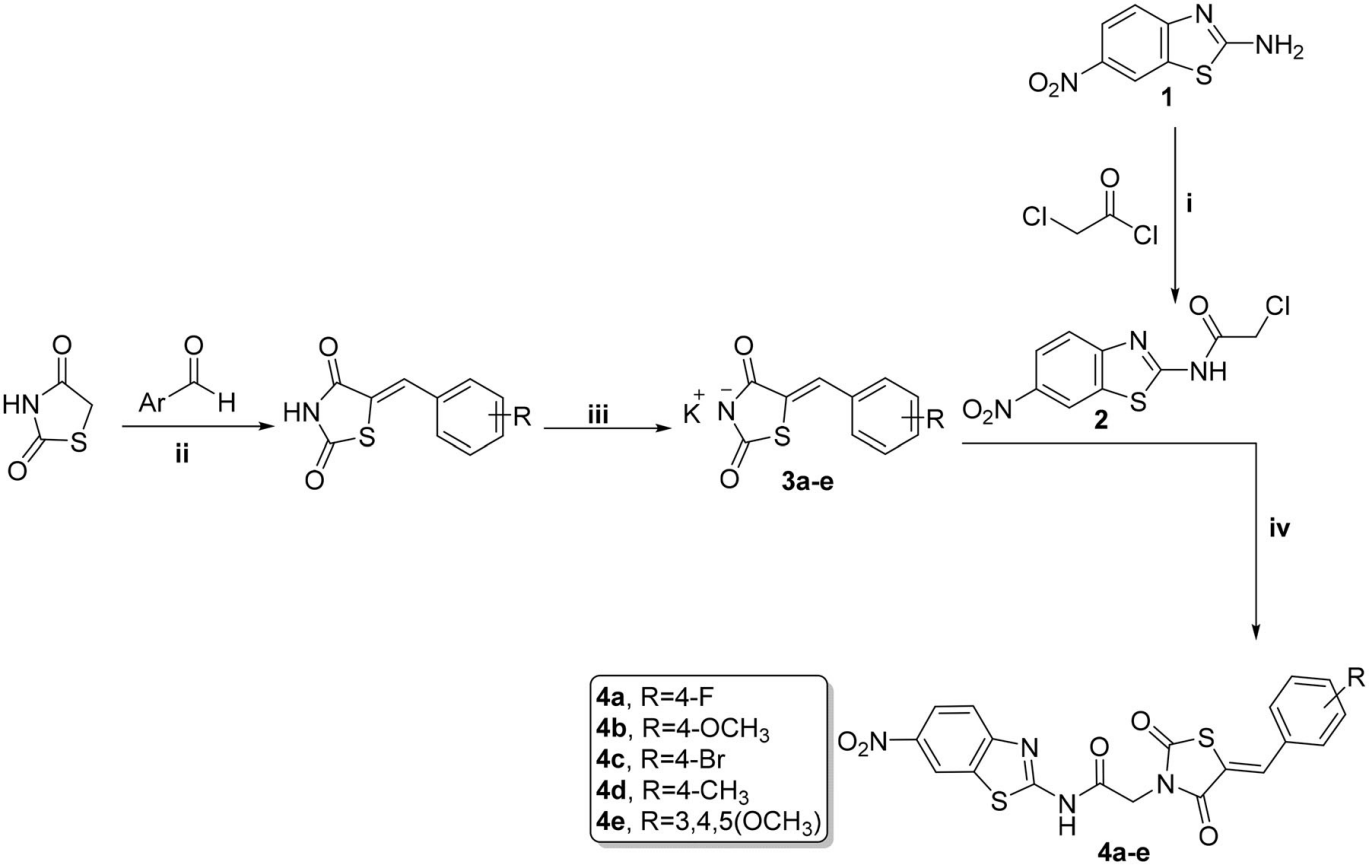
Preparation of benzothiazole/thiazolidine-2,4-dione hybrids **4a–e**; reagents and conditions: (i) CH_2_Cl_2_/Et_3_N 0 °C, rt; (ii) AcOH, NaOAc, reflux 14 h; (iii) EtOH, KOH, reflux 2 h; and (iv) DMF, K_2_CO_3_, reflux overnight.

**Scheme 2.**
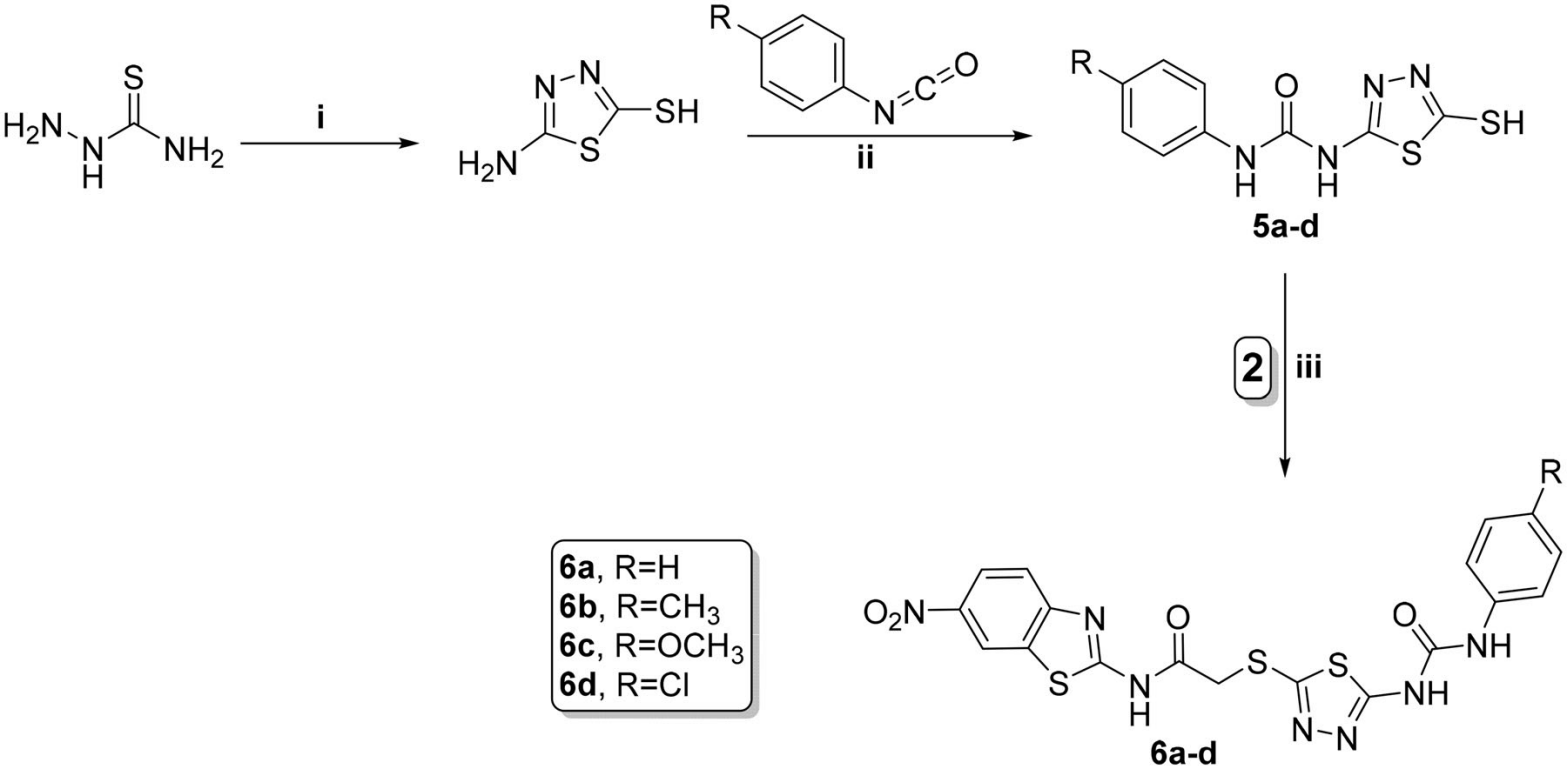
Synthesis of benzothiazole/1,3,4-thiadiazole-aryl urea hybrids **6a–d**; reagents and conditions: (i) CS_2_, EtOH, reflux overnight; (ii) CH_3_CN, reflux overnight; and (iii) acetone, K_2_CO_3_, reflux overnight.

**Scheme 3.**
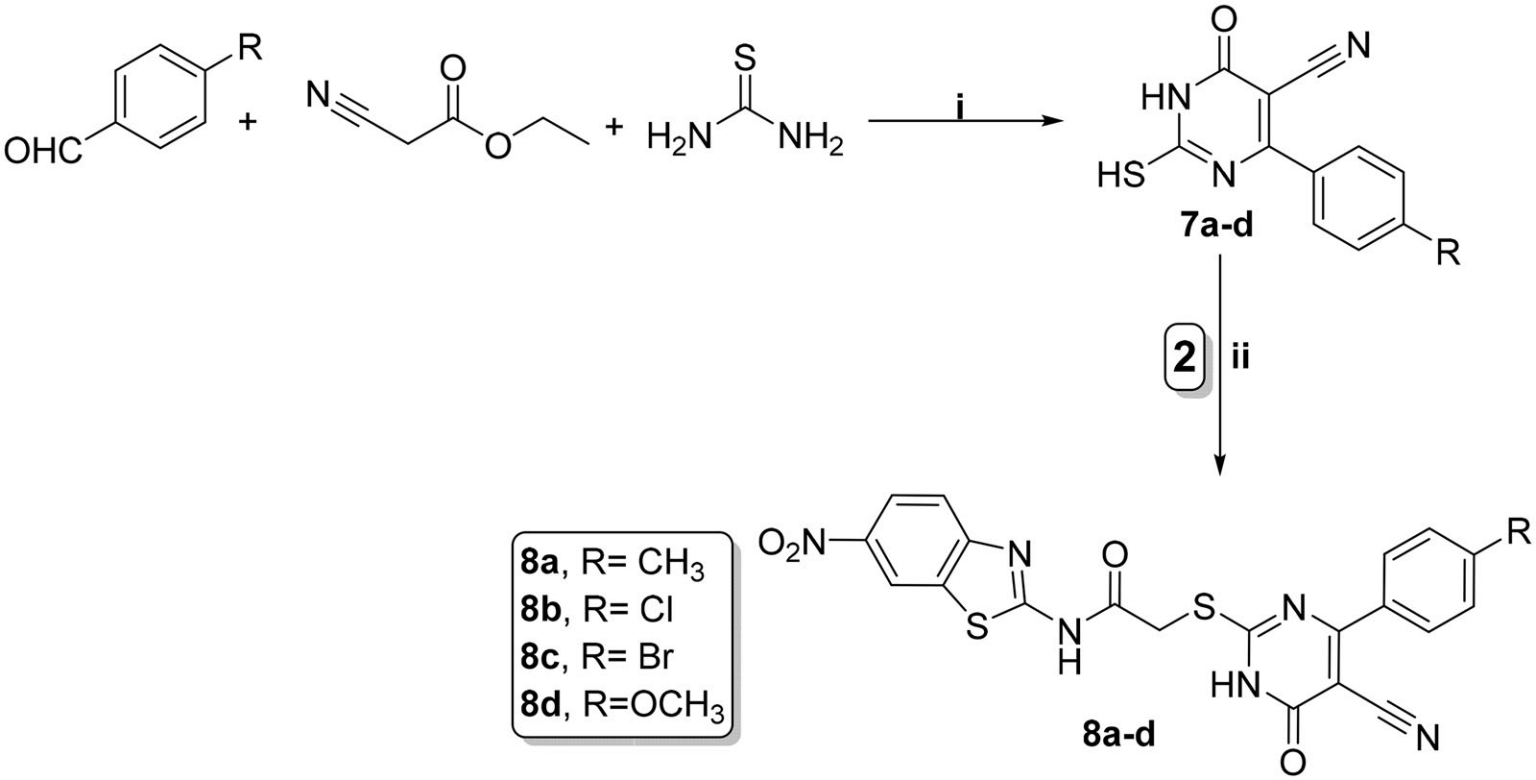
Synthesis of benzothiazole/cyanothiouracil hybrids **8a–d**; reagents and conditions: (i) EtOH, K_2_CO_3_, reflux 12 h and (ii) acetone, K_2_CO_3_, reflux overnight.

Synthesis of new analogs, thiazolidine-2,4-diones **4a–e**, 1,3,4-thiadiazoles **6a**–**d,** and cyanothiouracils **8a**–**d**, is illustrated in Schemes 1, 2, and 3. Starting with 6-nitrobenzo[*d*]thiazol-2-amine (**1)** which was subjected to acetylation using chloroacetyl chloride and triethylamine as a base, furnished the chloride derivative **2**, which is considered as the key precursor in the next nucleophilic substitution reactions^52^. 5-Arylidene-thiazolidine-2,4-diones were formed through a Knoevenagel condensation reaction, and then refluxed with ethanolic KOH to obtain the potassium salts **3a**–**f**, that were further substituted with the chloride derivative **2** to obtain the target benzothiazole/thiazolidine-2,4-dione hybrids **4a–e** (Scheme 1)^53^.

The key intermediates **5a–d** were obtained *via* refluxing the 2-thiol derivative with various 4-substituted phenyl isocyanate derivatives in acetonitrile^50^. The formed urea derivatives **5a–d** underwent reaction with the chloride derivative **2** yielding the target benzothiazole/thiadiazole-aryl urea hybrids **6a–d** (Scheme 2).

The 6-phenyl-2-thiouracil-5-carbonitrile derivatives **7a–d** were obtained by prolonged heating of different aldehydes, thiourea, and ethyl cyanoacetate with K_2_CO_3_ (Scheme 3)^54^. Finally, compounds **7a**–**d** were refluxed with the chloride derivative **2** in acetone using potassium carbonate to furnish the corresponding cyanothiouracils **8a**–**d** (Schemes 3).

The newly synthesised benzothiazole-based derivatives (**4a**–**e**, **6a–d,** and **8a–d**) were characterised using different means of IR, melting point, elemental analysis and spectroscopic data (^1^H-NMR, ^19^F-NMR, ^13^C-NMR, and MS). The IR charts of the new targets exhibited characteristic bands ranging from 1670 to 1730 cm^−1^ verifying the two amidic − C=O groups. ^1^H-NMR spectra for all the newly synthesised target compounds showed a characteristic singlet signal ranging from 4.3 to 4.8 ppm corresponding to methylene protons –N/SCH_2_CO– guaranteeing the synthesis of our new targets. In addition, ^13^C-NMR revealed the appearance of three peaks (**4a**–**e**) or two peaks (**6a–d** and **8a–d)** at ∼160–169 ppm equivalent to the carbonyl groups. Moreover, all final targets exhibited characteristic peak in the aliphatic region at approximately 37–44 ppm for the methyl carbon of acetamide moiety. All other data were consistent with the proposed structures. The mass spectra of target compounds showed the right molecular ion peaks (M^+^). The synthetic methods and the yields are presented in experimental part.

### Biological evaluation

#### In vitro cytotoxicity and antitumor activity:

The *in vitro* cytotoxic activity of the new benzothiazole hybrids **4a–e**, **6a**–**d,** and **8a**–**d** was evaluated by standard MTT assay against three human cancer cell lines: HCT-116 (colorectal carcinoma), HEPG-2 (hepatocellular carcinoma), and MCF-7 (breast cancer) cell lines^55^, using SOR as a positive drug control. IC_50_ values were calculated and summarised in Table 1.

**Table 1.** Cytotoxicity (IC_50_) of the target hybrids **4a–e**, **6a**–**d,** and **8a**–**d** towards HCT-116, HepG-2, MCF-7, and WI-38 cell lines.

		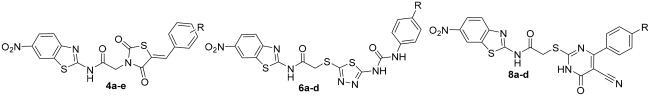			
Comp.	R	IC_50_ (µM)^a^			
		HCT-116	HepG-2	MCF-7	WI-38
**4a**	4-F	5.61 ± 0.3	7.92 ± 0.6	3.84 ± 0.1	45.23 ± 2.6
**4b**	4-OCH_3_	29.92 ± 2.2	34.68 ± 2.4	22.27 ± 1.7	77.57 ± 4.3
**4c**	4-Br	47.94 ± 2.9	65.47 ± 3.6	50.05 ± 2.9	28.85 ± 2.2
**4d**	4-CH_3_	26.78 ± 2.1	21.13 ± 1.8	15.48 ± 1.2	74.01 ± 4.1
**4e**	3,4,5-(OCH_3_)_3_	8.24 ± 0.6	9.04 ± 0.8	6.11 ± 0.4	32.81 ± 2.3
**6a**	H	92.45 ± 4.9	83.20 ± 4.4	72.62 ± 3.9	57.84 ± 3.5
**6b**	4-CH_3_	41.68 ± 2.7	52.33 ± 3.1	39.79 ± 2.3	86.20 ± 4.8
**6c**	4-OCH_3_	80.82 ± 4.3	76.90 ± 4.0	67.38 ± 3.8	49.11 ± 2.8
**6d**	4-Cl	64.41 ± 3.6	69.26 ± 3.8	58.61 ± 3.2	85.66 ± 4.5
**8a**	4-CH_3_	13.89 ± 1.0	18.10 ± 1.4	10.86 ± 0.8	53.10 ± 3.1
**8b**	4-Cl	54.06 ± 3.2	36.98 ± 2.5	43.13 ± 2.5	>100
**8c**	4-Br	38.29 ± 2.5	27.94 ± 2.2	31.30 ± 2.1	>100
**8d**	4-OCH_3_	>100	87.52 ± 4.7	78.22 ± 4.1	63.24 ± 3.8
**SOR**	–	5.23 ± 0.3	4.50 ± 0.2	4.17 ± 0.2	6.72 ± 0.5

^a^IC_50_ is defined as the concentration needed to inhibit 50% of cancer cell proliferation. Data are represented as the mean ± SD from the dose-response curves as triplicate . IC_50_, (µg/mL): 1–10 (very strong), 11–20 (strong), 21–50 (moderate), 51–100 (weak), 100–200 (very weak), above 200 (non-cytotoxic).

The results showed that the investigated derivatives displayed different degrees of cytotoxic effect against the tested cancer cell lines.

#### Structure–activity correlation

The IC_50_ results of benzothiazole/thiazolidine-2,4-dione hybrids **4a–e** revealed that compounds **4a** and **4e** showed wide spectrum cytotoxic potencies and are considered the most potent members in this series with IC_50_ values range of 3.84–9.04 μM towards the three cell lines. **4a** is considered the best member of all compounds with IC_50_ of 5.61, 7.92, and 3.84 μM against HCT-116, HEPG-2, and MCF-7, respectively, compared to SOR (IC_50_ of 5.23, 4.50, and 4.17 μM, respectively), while, compounds **4d** and **4b** exhibited moderate cytotoxicity against the three cell lines with IC_50_ of 26.78, 21.13, and 15.48 for compound **4d** and IC_50_ of 29.92, 34.68, and 22.27 for compound **4b** against HCT-116, HEPG-2, and MCF-7 cell lines. In addition, compound **4c** displayed a diminished activity with IC_50_ range of 47.94 to 65.47 compared to the other analogs.

It is observed that the chemistry of hybrids **4a–e** influenced their anticancer activity. Compound **4a** with small electron withdrawing substituent (F) on *para* position of benzylidene group showed higher antitumor activity (IC_50_ range of 3.84–5.61 μM) than compound **4c** which contains a bulky substituent (Br) (IC_50_ range of 47.94–65.47 μM). However, the introduction of electron donating substituent on *para* position (OCH_3_, CH_3_) as in compounds **4b** and **4d** resulted in a drop of the antitumor activity (IC_50_ range of 22.27–34.68 and 15.48–26.78 μM, respectively). Moreover, the 3,4,5-trimethoxy substitution resulted in a sharp increase in the antitumor activity as in compound **4e** with IC_50_ of 6.11– 9.04 μM.

Regarding IC_50_ values of benzothiazole/1,3,4-thiadiazole-aryl urea hybrids **6a–d**, they generally revealed a much weaker cytotoxic activity towards the examined cell lines (IC_50_ range: 39.79–92.45 μM) compared to thiazolidine-2,4-dione **4** and cyanothiouracil **8** hybrids.

The benzothiazole/cyanothiouracil hybrids **8a–d** showed a variable degree of cytotoxic potencies. Compound **8a** is considered the most active member in this series with IC_50_ of 13.89, 18.10, and 10.86 μM against HCT-116, HEPG-2, and MCF-7, respectively.

**8a** (IC_50_ of 10.86–18.10 μM) with small π-rich substituent (CH_3_) on *para* position of phenyl group had the highest activity against the three cell lines than those containing bulky substituent (OCH_3_) as in compound **8d** (IC_50_ of 78.22 - >100 μM). On the other hand, the introduction of π-deficient *para* substituted phenyl moiety (Cl and Br) decreased the activity as in compounds **8b** and **8c** with IC_50_ range of 27.94–54.06 μM).

#### In vitro cytotoxicity against human normal cell

**Figure 4.**
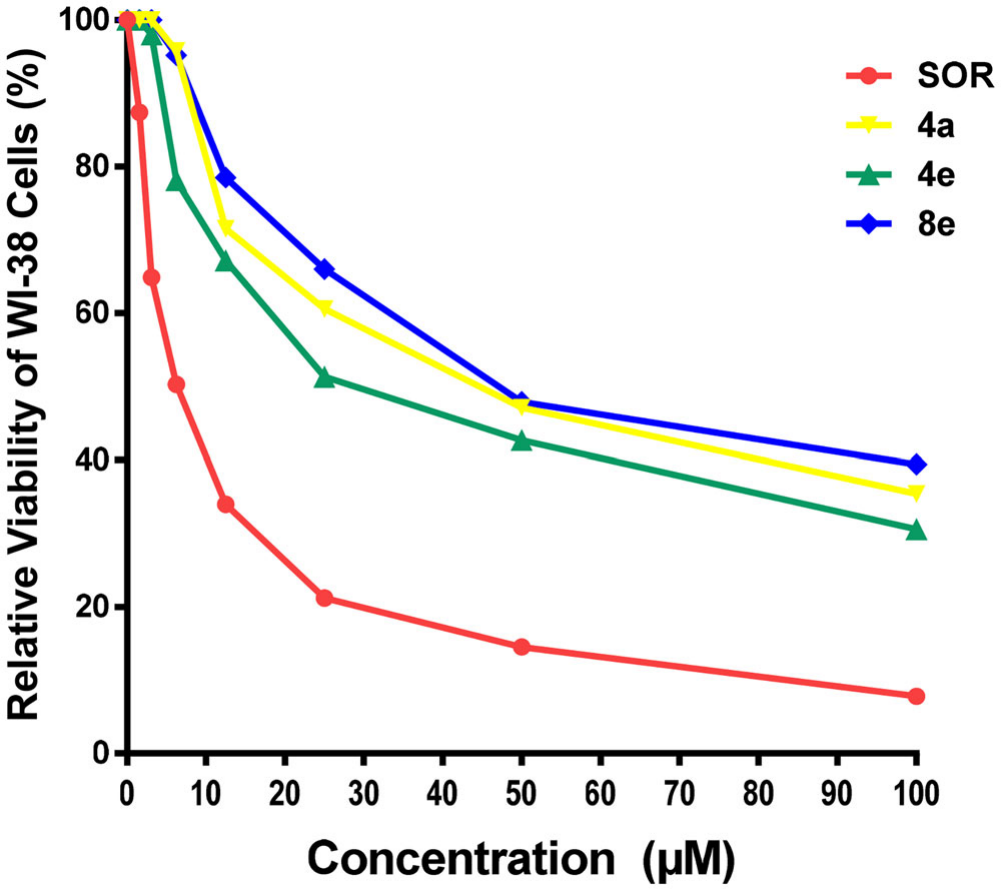
Cytotoxic effect of the most active compounds **4a, 4e, 8e,** and **SOR** on human normal WI-38 cell line.

All tested compounds were studied for their cytotoxic activity on the WI-38 normal fibroblast WI-38 cells to assess their therapeutic safety. As depicted in Table 1, all the investigated hybrids showed lower toxicity against normal fibroblast cells WI-38. Accordingly, compounds **4a**, **4e,** and **8a** exhibited low cytotoxicity on WI-38 with IC_50_ values of 45.23, 32.81, and 53.10 μM, respectively in comparison to SOR (IC_50_ = 6.72 μM) (Figure 4).

#### VEGFR-2 enzyme inhibition assay

The promising hybrids, displaying the highest anticancer potencies were selected to further assess their dose-dependent VEGFR-2 enzyme inhibition at four various concentrations (10 nM–100 nM–1 μM–10 μM) to determine their IC_50_ values. As depicted in Table 2, all tested compounds had high % inhibition values against VEGFR-2 ranging from 70.11 to 77.94% at concentration of 1 μM, and range of 83.47–91.96% at concentration of 10 μM which is very close to the standard drug SOR. At concentration of 10 μM, **4a** showed VEGFR-2% inhibition of 91.96% which is very close to the standard drug used (92.17%).

**Table 2.** Inhibitory effects of compounds **4a, 4e,** and **8a** against VEGFR-2.

Comp.	% Inhibition				IC_50_ (nM)
	10 nm	100 nm	1 μM	10 μM	
**4a**	31.27	45.14	77.94	91.96	91
**4e**	24.09	42.84	70.45	88.69	161
**8a**	16.31	37.14	70.11	83.47	266
**SOR**	32.47	57.22	81.01	92.17	53

In addition, consistent with the cytotoxic activity, compound **4a** showed strong VEGFR-2 inhibitory activity with IC_50_ of 91 nM, in comparison with 53 nM for SOR. The benzothiazole derivative **4e** exhibited lower inhibitory effect with IC_50_ of 161 nM. Also, the IC_50_ of compound **8a** was 266 nM which could explain the moderate cytotoxic activity observed in MTT assay. It is worth mentioning that compound **4a** (IC_50_ of 91 nM), with 6-nitrobenzothiazole and F atom on *para* position of benzylidene group, was more potent than the corresponding non-nitrated benzothiazole derivative **X** with IC_50_ of 190 nM^45^.

#### Cell cycle analysis

For further investigation of the mode of antitumor activity of the most potent candidates **4a**, **4e,** and **8a**, cell cycle analysis and apoptosis induction were done using propidium iodide (PI) staining^67^. MCF-7 breast cancer cells were incubated with IC_50_ concentration of compounds **4a**, **4e,** and **8a** for 24 h, stained with PI and DNA contents were measured by flow cytometry (FCM). The cells were treated with DMSO as a control.

**Figure 5.**
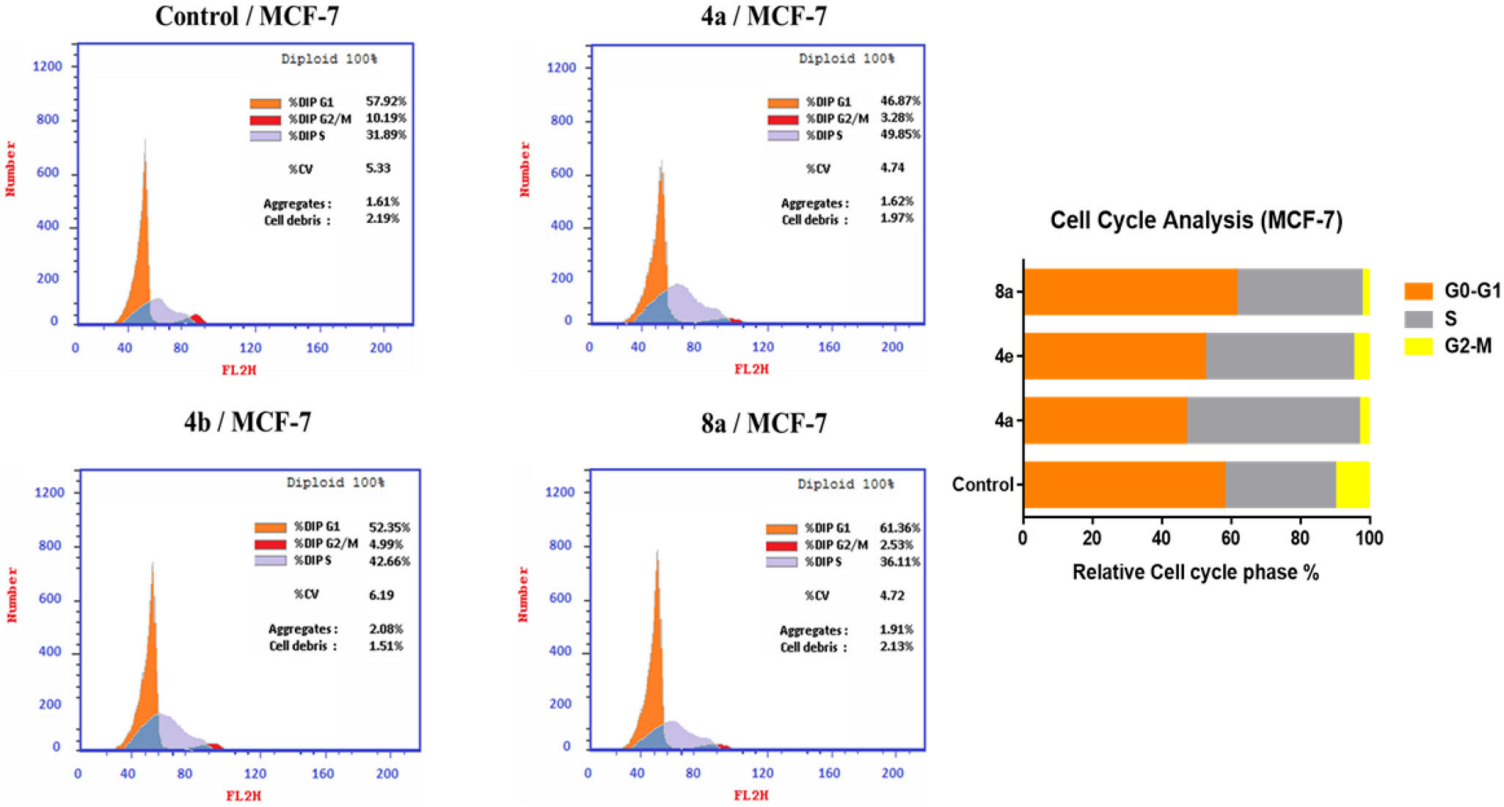
Effect of compounds **4a**, **4e,** and **8a** on DNA-ploidy flow cytometric analysis of MCF-7 cells. The cells were treated with DMSO as control and **4a**, **4e,** and **8a** for 24 h.

Quantification of the results (Table 3, Figure 5) showed that compounds **4a** and **4e** arrested MCF-7 cells at S phase. The cell population in S phase increased from 31.89 in the untreated cell to 49.85 and 42.66% in the cell treated with compounds **4a** and **4e**, respectively. On the other hand, compound **8a** led to an outstanding growth in ratio of MCF-7 cells in G0/G1 phase from 57.92% in the untreated cells to 61.36% and in S phase from 31.89% in the untreated cells to 36.11% with a decrease in the ratio of cells in G2/M phase by 2.53% in comparison with untreated control (10.19%). These findings obviously show that **8a** arrested the cell cycle in MCF-7 at a G1/S phase.

**Table 3.** Effect of compounds **4a**, **4e,** and **8a** on the cell cycle progression in MCF-7 cells compared to SOR.

Comp.	Cell cycle distribution (%)		
	G0-G1	S	G2-M
**4a**	46.87	49.85	3.28
**4e**	52.35	42.66	4.99
**8a**	61.36	36.11	2.53
**Control (DMSO)**	57.92	31.89	10.19

#### Detection of apoptosis

**Figure 6.**
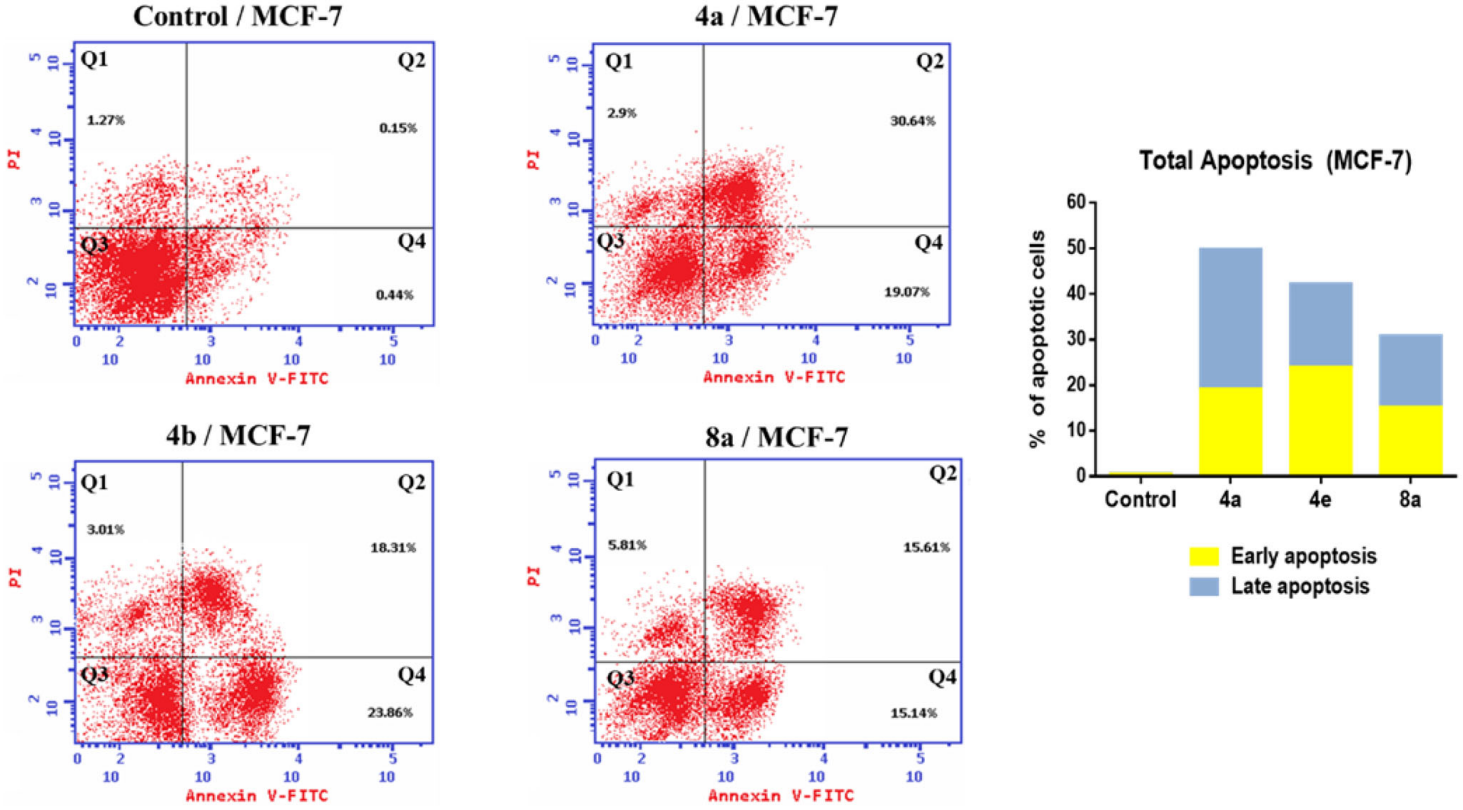
Effect of compounds **4a**, **4e,** and **8a** on the percentage of annexin V-FITC-positive staining in MCF-7 cells. The cells were treated with DMSO as control and **4a**, **4e,** and **8a** for 24 h. Q1 quadrant represents dead (necrotic) cells; Q2 quadrant represents late apoptosis; Q3 quadrant represent live cells; Q4 quadrant represents early apoptosis.

The development of apoptosis inducers is highly attractive therapeutic strategy for the identification of powerful anticancer drugs^67^^,^^68^. So that, the ability of the most active compounds **4a**, **4e,** and **8a** to induce apoptosis in MCF-7 cells was evaluated using AnnexinV and PI double staining flow cytometry. As shown in Figure 6, MCF-7 cells treated with compounds **4a**, **4e,** and **8a** exhibited the accumulation of early and late apoptotic cells, as compared to the control. For example, the ratio of total apoptotic cells (early and late apoptotic cells) after treatment of MCF-7 cells with compounds **4a**, **4e,** and **8a** for 24 was sharply increased to 49.71, 42.17, and 30.75%, respectively as compared to 0.59% of apoptotic cells in the untreated control. These results indicated that these compounds could inhibit cell growth through induction of cell apoptosis.

### In silico evaluation and ADME prediction

#### Molecular docking results

**Figure 7.**
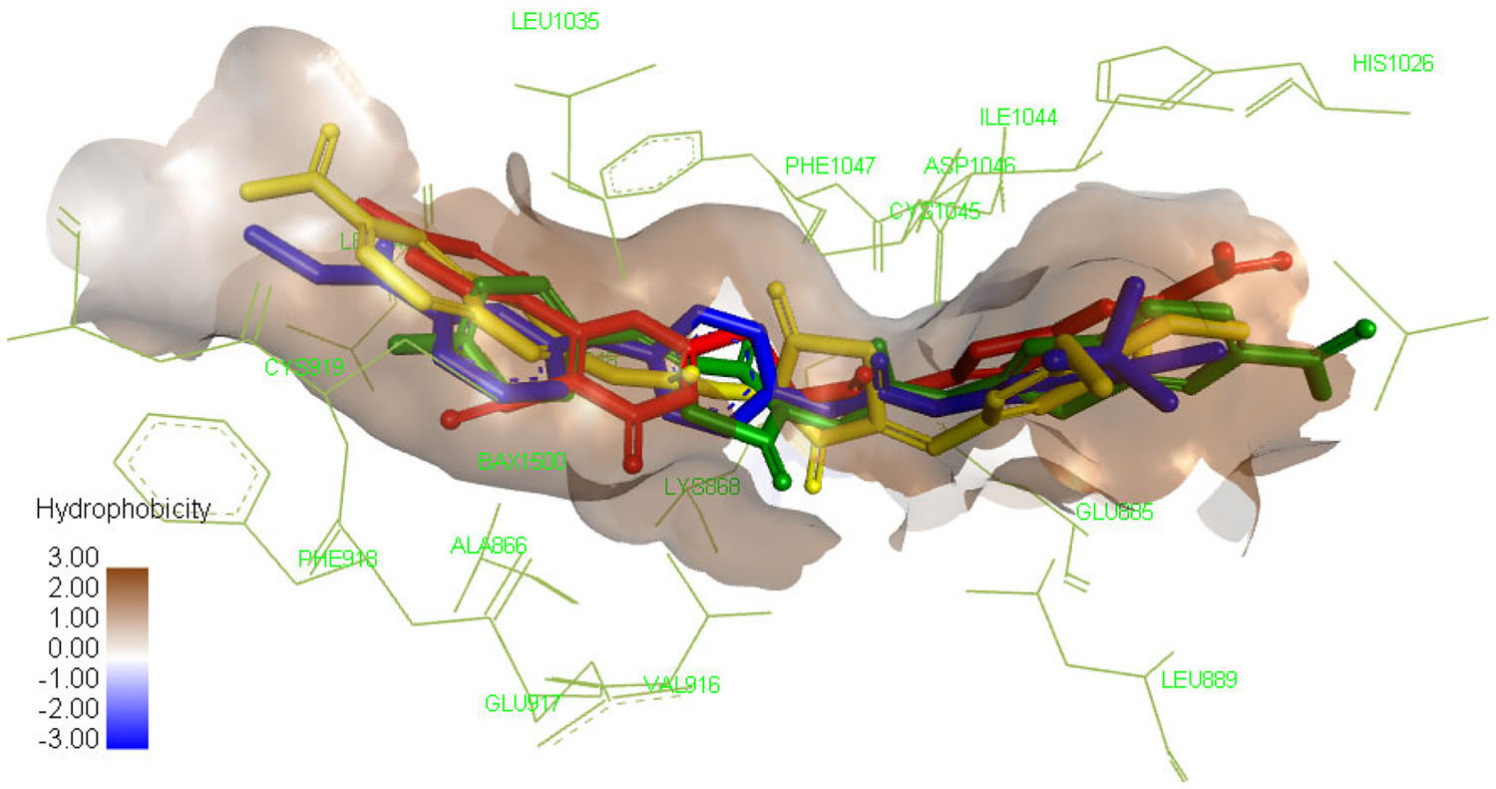
Compound **4a** compound **4e** compound **8a** docked in the active site and aligned to the co-crystallized ligand SOR. Please refer to the online version for colored figure.

Molecular docking proved to be a versatile tool in the field of drug discovery due to its ability to explain the interaction of small molecules with various biological targets accelerating the discovery and development of new potential therapeutics^69–74^. Since VEGFR-2 is one of the most investigated targets using *in silico* tools, Molecular docking was preformed to identify the interaction of compounds **4a, 4e,** and **8a** with the active site. All the three compounds were able to occupy the same active site of SOR, the co-crystallized ligand as depicted in Figure 7 but showed different interaction profile with the binding site. Compound **4a** showed the lowest binding energy followed by compound **4e** and **8a**, respectively as shown in Table 4.

**Table 4.** Binding energy of the top three compounds docked in VEGFR-2 (PDB: 4ASD) in comparison to the co-crystallized ligand SOR.

Compound	FlexXscore
**4a**	−24.1
**4e**	−20
**8a**	−17.9
**SOR**	−35.6

Concerning compound **4a**, interaction with important residues in the active site was achieved through hydrogen bonding such as Asp1046 through its carbonyl group and Glu885 through the NH in the amide group which is a known requirement to achieve good inhibitory activity against this enzyme. In addition, certain hydrophobic interactions have been achieved with IL888, ILE892, Leu1019, ILE1025, and His1026 residues. Furthermore, the nitro group of benzothiazole was able to form hydrogen bond with Ile1025, and ionic bond with ASP814 and Arg1027 which could explain the superior activity of our compound **4a** over the previously reported non-nitrated derivative **X**^45^. The thiazolidine-2,4-dione moiety was found to occupy the linker area in the active site interacting with Lys868, Val 899, Val916, and Phe1047 allowing the fluoro-phenyl ring to interact with several residues in the hydrophobic groove such as Leu840, Ala866, and Leu1035 through hydrophobic interactions and with Cys919 through halogen hydrogen bond as demonstrated in Figure S1. These extensive interactions with the active site may explain its ability to inhibit the enzyme experimentally at low nanomolar concentration.

Despite the structural similarity between compounds **4a** and **4e**, the later showed different binding pattern, as it was only able to form one hydrogen bond with Asp1046 but not with Glu885 which explains the significant increase of IC_50_ shown in the experimental enzyme inhibition assay as this interaction is important for achieving potent inhibitory activity. Still, this was compensated by the interaction of the compound with several amino acid residues in the active site through hydrophobic interaction of benzothiazole ring with Ala866, Leu840, Phe918, Cys919, and Leu1035, while the thiazolidine-2,4-dione formed hydrophobic interaction with Val899, Val916, Cys1045, and Lys868. Finally, the trimethoxy substituted phenyl occupied the hydrophobic allosteric site so it was able to interact with Ile892, Val898, Ile1044, and Leu1019 as demonstrated in Figure S2.

Regarding compound **8a**, it showed similar binding pattern to that of compound **4a** since it was able to form two hydrogen bonds between the amide moiety and Asp1046 and Glu885. Still, it protrudes towards the ATP active site allowing the interaction of cyanothiouracil with Cys919 through the cyanide group and with Leu840, Val 899, Ala866, Cys919, Phe1047, Leu1035, and Cys1045 through hydrophobic interactions. Consequently, the benzothiazole ring was not able to fully occupy the hydrophobic pocket in the allosteric site, so it interacted only with His1026 (Figure S3) which explains the moderate inhibitory activity demonstrated in the experimental enzyme inhibition assay.

#### ADMET analysis

Predicting the pharmacokinetic characteristics (ADMET) is important in early stage of drug development to enhance the efficacy and safety profile and to avoid the failure of therapeutic agents to be effective clinical candidates^75^. Thus, the ADMET profile of the most active hybrids **4a**, **4e,** and **8a** was calculated theoretically using online Pre-ADMET server^76^.

As shown in Table 5, the obtained results revealed that all the tested hybrids predicted to have negative carcinogenic activities and exerted excellent intestinal absorption with HIA values ranging from 83.30 to 91.96%, demonstrating their potency as oral anticancer candidates. They also showed low CNS absorption, low cell permeability in the CaCo2 cell model (CaCo2 values < 4 nm/s) and low cell permeability in the MDCK cell model (MDCK < 25 nm/s). Moreover, all hybrids are expected to be not incorporated in drug-drug interactions as they are non-inhibitors of CYP3A4 enzyme, in contrast to reference drug which has CYP3A4 inhibitory activity. Similarly, as reference drug, all the hybrids are inhibitors to P-glycoprotein (PgP), increasing bioavailability of co-administered drugs and reversing multi-drug resistance in cancer cells.

**Table 5.** ADMET profile for active compounds **4a**, **4e**, **8a,** and **SOR**.

Comp.	HIA	CaCo2	MDCK	BBB	CYP3A4 inhibition	PgP inhibition	Carcinogenicity
**4a**	91.96	0.67	0.13	0.11	Non	Inhibitor	Negative
**4e**	86.05	3.06	0.08	0.07	Non	Inhibitor	Negative
**8a**	83.30	0.44	0.13	0.07	Non	Inhibitor	Negative
**SOR**	93.50	22.55	0.08	1.00	inhibitor	Inhibitor	Negative

*Notes:* HIA: human intestinal absorption (%), CaCo2: permeability through cells derived from human colon adenocarcinoma (nm/s), MDCK: permeability through Maden Darby canine kidney cells (nm/s); tool for rapid permeability, BBB: blood brain barrier penetration, CYP3A4: cytochrome P450 3A4, PgP: P-glycoprotein.

## Conclusion

In summary, three series of novel benzothiazole-based hybrids bearing thiazolidine-2,4-dione **4a–e**, thiadiazole-aryl urea **6a–d** and cyanothiouracil **8a–d** were synthesised, their structure was confirmed and evaluated for their cytotoxic activity against three human cancer cell lines: HCT-116, HEPG-2, and MCF-7. Thereafter, the most effective anti-proliferative members were explored for their VEGFR-2 inhibitory activity. Compounds **4a**, **4e,** and **8a** were the most active hybrids. Hybrid **4a** was found to be the most active one with wide spectrum of cytotoxic activity against the three cell lines with IC_50_ of 5.61, 7.92, and 3.84 µM, respectively. The most effective Hybrids **4a**, **4e,** and **8a** also displayed an evident apoptosis in MCF-7 cell lines and induced cell cycle arrest at the S phase (compounds **4a** and **4e**) and at G1/S phase (compound **8a**). Consistent with the cytotoxic activity, compound **4a** showed strong VEGFR-2 inhibition with IC_50_ 91 nM, in comparison with 53 nM for SOR. The cytotoxic and VEGFR-2 inhibition activities were illustrated using molecular docking study which revealed that **4a** involves the key fragment important for the interaction with the DFG-binding domain that is essential for VEGFR-2 hindrance. *In silico* study involving ADMET analysis were performed where compound **4a** showed acceptable physicochemical and pharmacokinetic properties. Still, further chemical optimisation and pharmacokinetic studies are needed before considering this class of compounds as potential candidate for the development of future anticancer agents.

## Supplementary Material

Supplemental Material
