# Supplementary material for: New benzothiazole hybrids as potential VEGFR-2 inhibitors: design, synthesis, anticancer evaluation, and in silico study
Source: J Enzyme Inhib Med Chem. 2023 Jan 24;38(1):2166036. doi: 10.1080/14756366.2023.2166036 (PMC9879182; doi:10.1080/14756366.2023.2166036)
Supplement: Supplemental Material [file IENZ_A_2166036_SM9242.pdf]

Supplementary file

New benzothiazole hybrids as Potential VEGFR-2 inhibitors: Design, Synthesis, Anticancer Evaluation and *In Silico* Study

Mohammad M. Al-Sanea[1,†,*], Abdelrahman Hamdi[2,†], Ahmed A. B. Mohamed[3,†,*], Hamed W. El-Shafey[2], Mahmoud Moustafa[4], Abdullah A. Elgazar[5], Wagdy M. Eldehna[6], Hidayat Ur Rahman[7], Della G. T. Parambi[1], Rehab M. Elbargisy[8], Samy Selim[9], Syed Nasir Abbas Bukhari[1], Omnia Magdy Hendawy[10], Samar S. Tawfik[2]

Supplementary figures

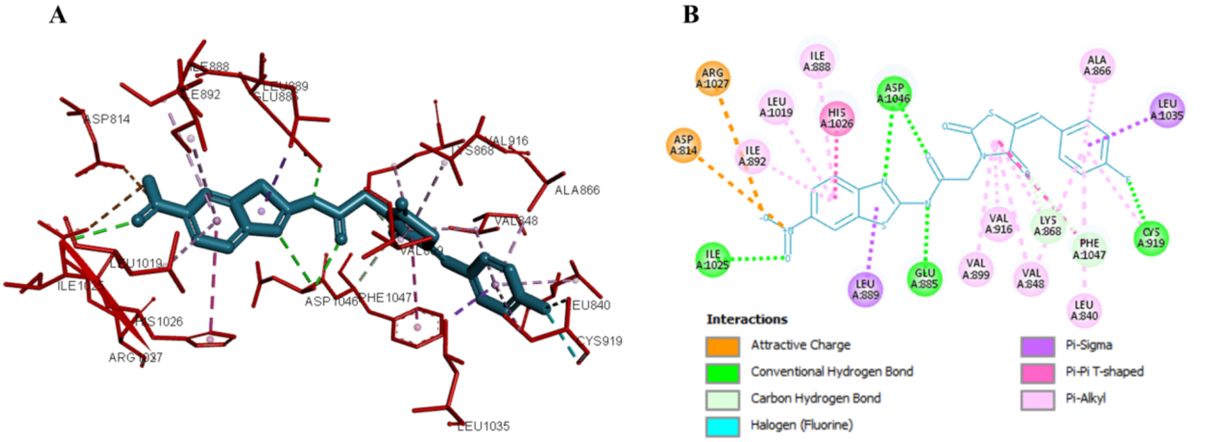

Fig. S1. A) 3D interactions of **4a** (Turquoise) with the active site of VEGF2 **B)** 2D interactions of **4a** with amino acids in the active site of VEGFR-2.

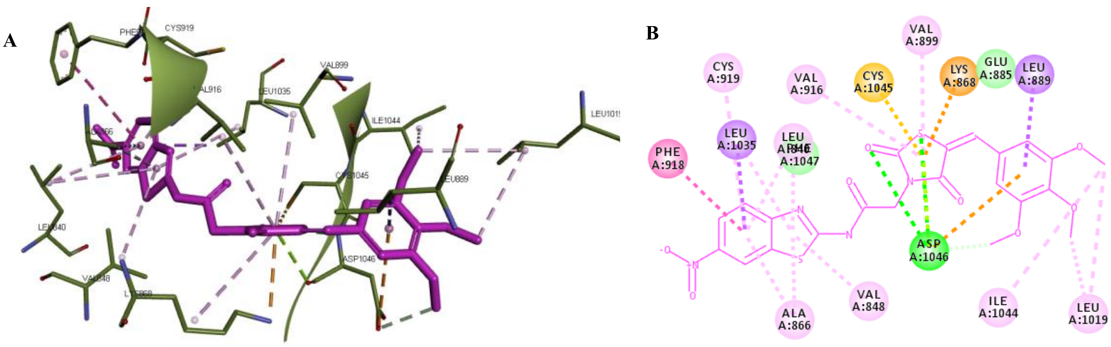

Fig. S2. A) 3D interactions of **4e** (magenta) with the active site of VEGFR-2. **B)** 2D interactions of **4e** with amino acids in the active site of VEGFR-2.

A **B**

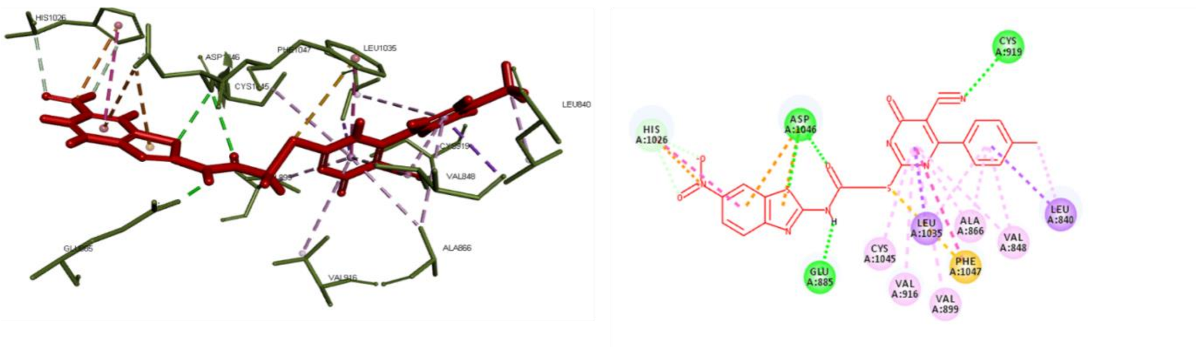

Fig. S3. A**)** 3D interactions of **8a** (Red) with the active site of VEGFR-2. **B)** 2D interactions of **8a** with amino acids in the active site of VEGFR-2.

NMR Charts

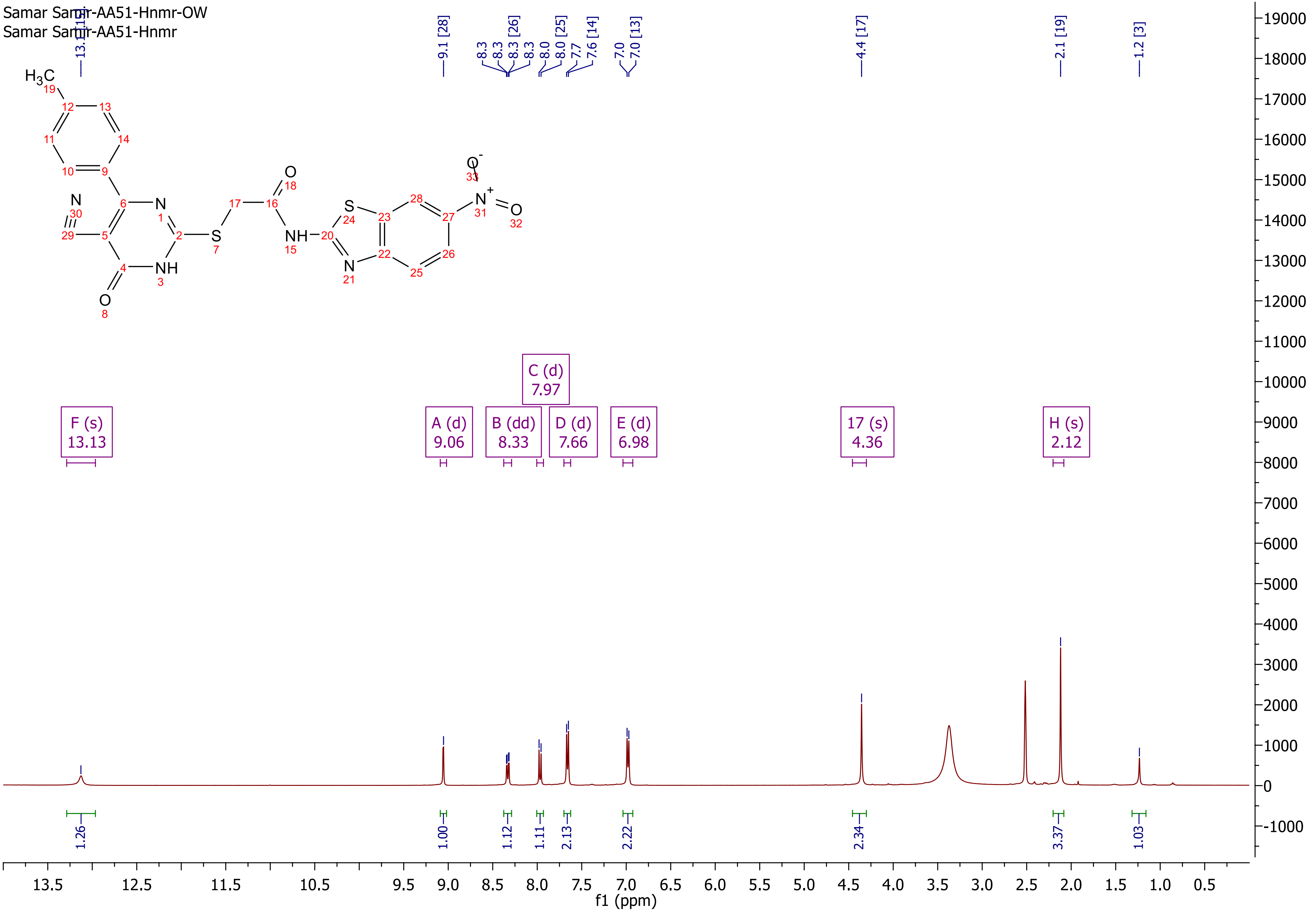

Samar Samir-AA51-Hnmr-OW
Samar Samir-AA51-Hnmr
13.1 [15]
9.1 [28]
8.3
8.3
8.3 [26]
8.3
8.0
8.0 [25]
7.7
7.6 [14]
7.0
7.0 [13]
4.4 [17]
2.1 [19]
1.2 [3]
F (s) 13.13
A (d) 9.06
B (dd) 8.33
C (d) 7.97
D (d) 7.66
E (d) 6.98
17 (s) 4.36
H (s) 2.12
1.26
1.00
1.12
1.11
2.13
2.22
2.34
3.37
1.03
f1 (ppm)

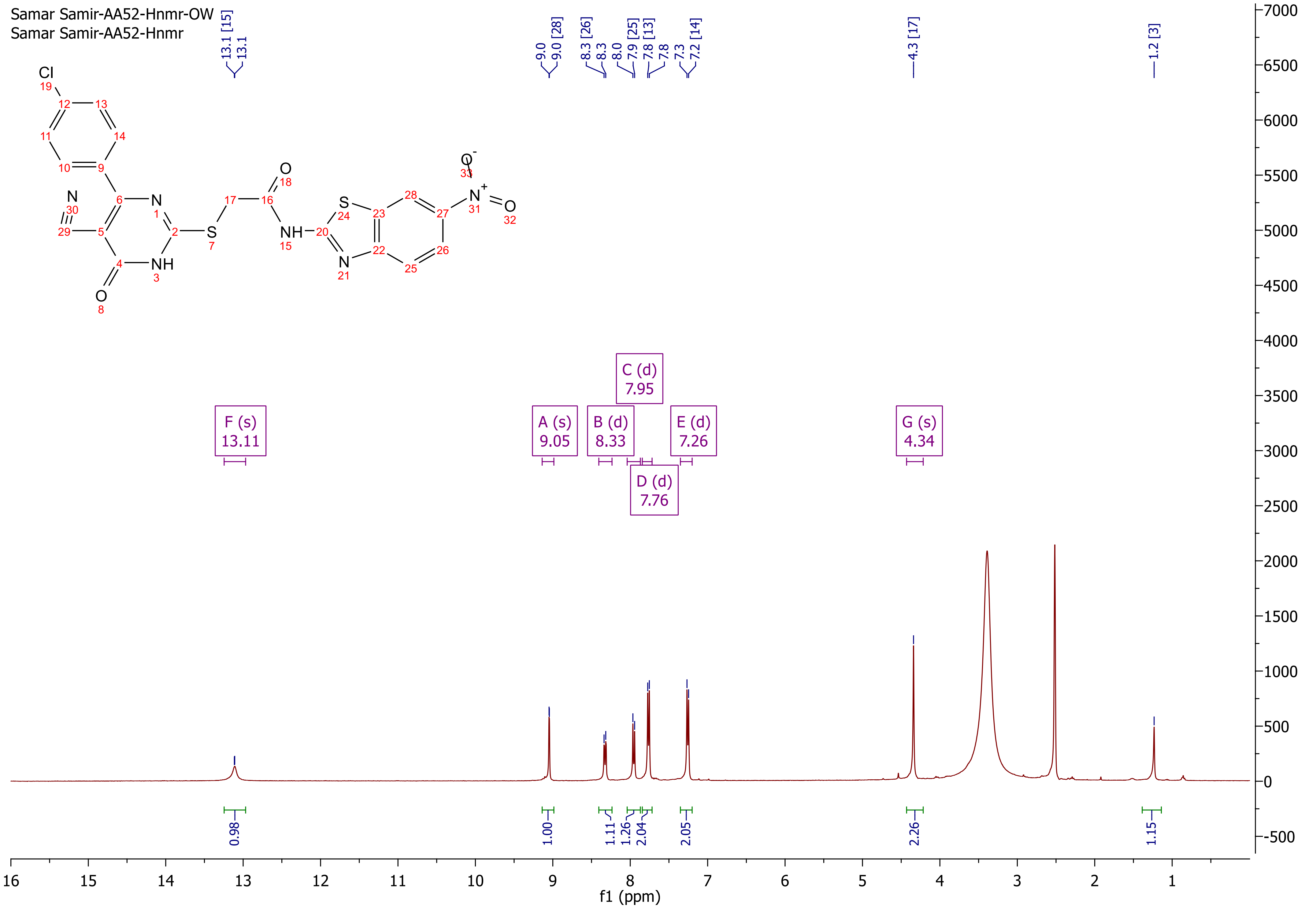

Samar Samir-AA52-Hnmr-OW
Samar Samir-AA52-Hnmr
13.1 [15]
13.1
9.0
9.0 [28]
8.3 [26]
8.3
8.0
7.9 [25]
7.8 [13]
7.8
7.3
7.2 [14]
4.3 [17]
1.2 [3]
F (s) 13.11
A (s) 9.05
B (d) 8.33
C (d) 7.95
D (d) 7.76
E (d) 7.26
G (s) 4.34
0.98
1.00
1.11
1.26
2.04
2.05
2.26
1.15
f1 (ppm)

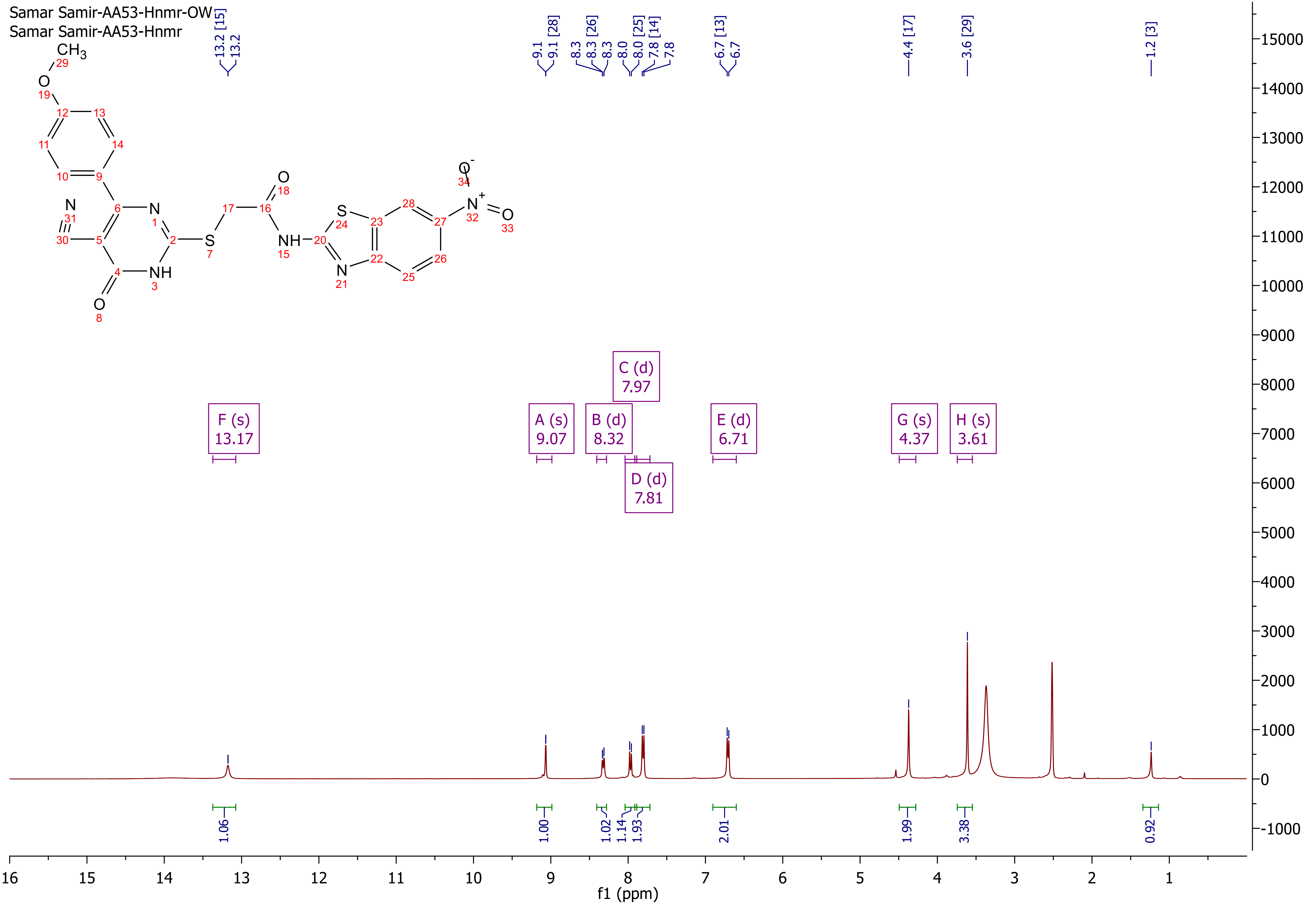

Samar Samir-AA53-Hnmr-OW
Samar Samir-AA53-Hnmr
13.2 [15]
13.2
9.1
9.1 [28]
8.3
8.3 [26]
8.3
8.0
8.0 [25]
7.8 [14]
7.8
6.7 [13]
6.7
4.4 [17]
3.6 [29]
1.2 [3]
F (s) 13.17
A (s) 9.07
B (d) 8.32
C (d) 7.97
D (d) 7.81
E (d) 6.71
G (s) 4.37
H (s) 3.61
1.06
1.00
1.02
1.14
1.93
2.01
1.99
3.38
0.92
f1 (ppm)

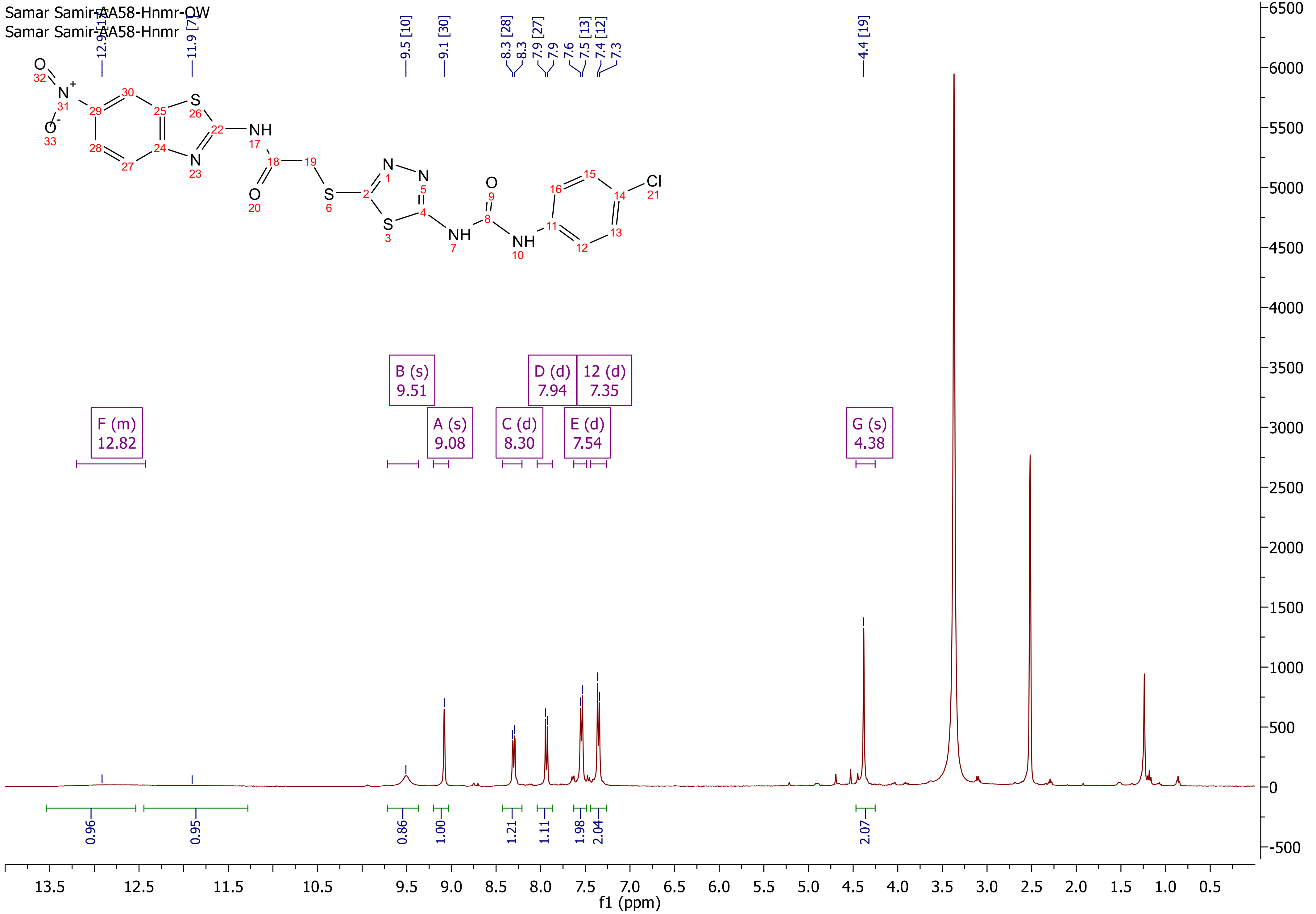
Samar Samir-AA58-Hnmr-OW
Samar Samir-AA58-Hnmr
12.9 [17]
11.9 [7]
9.5 [10]
9.1 [30]
8.3 [28]
8.3
7.9 [27]
7.9
7.6
7.5 [13]
7.4 [12]
7.3
4.4 [19]
B (s) 9.51
D (d) 7.94
12 (d) 7.35
F (m) 12.82
A (s) 9.08
C (d) 8.30
E (d) 7.54
G (s) 4.38
0.96
0.95
0.86
1.00
1.21
1.11
1.98
2.04
2.07
f1 (ppm)

Samar Samir-AA59-Hnmr-OW
Samar Samir-AA59-Hnmr

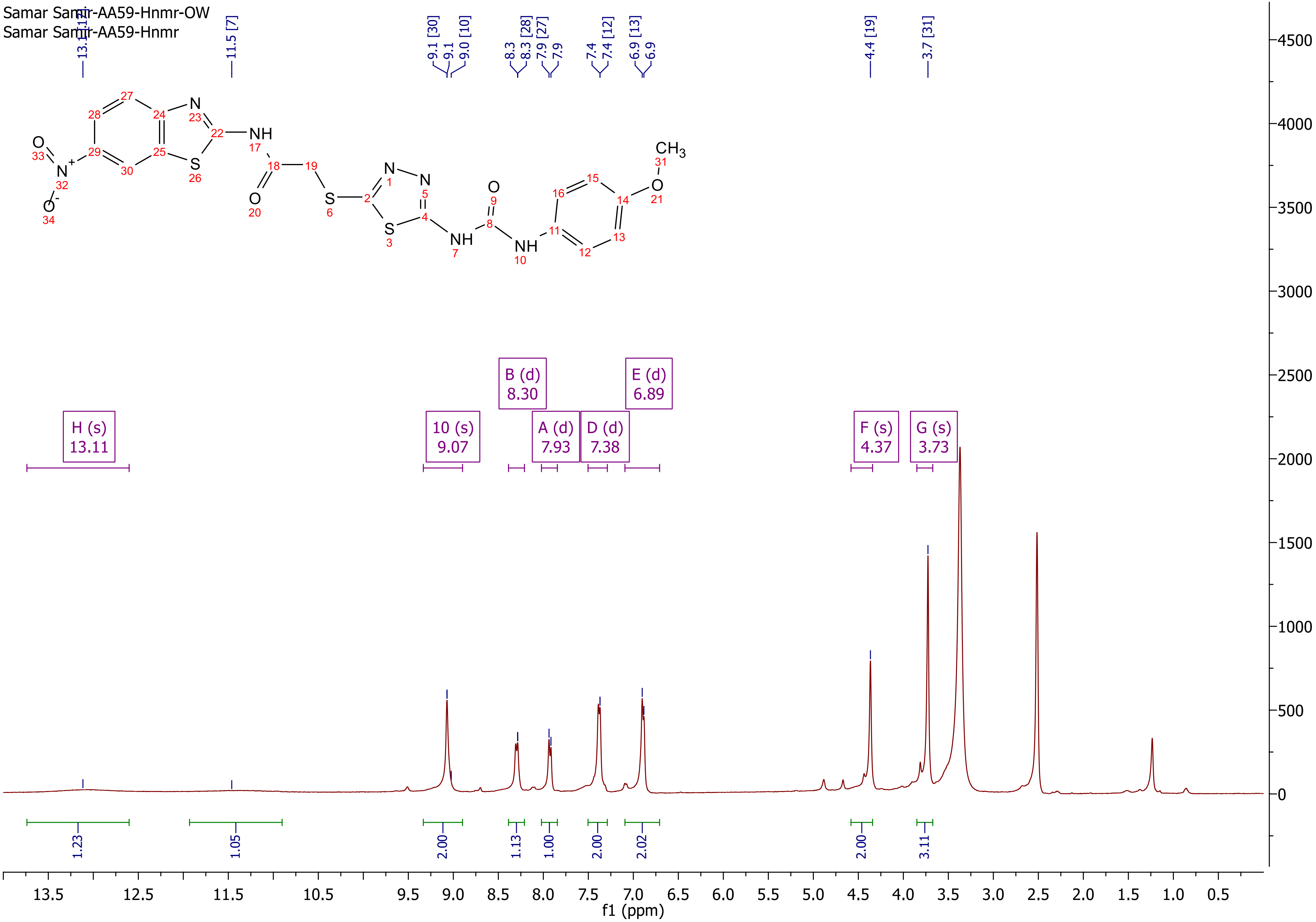

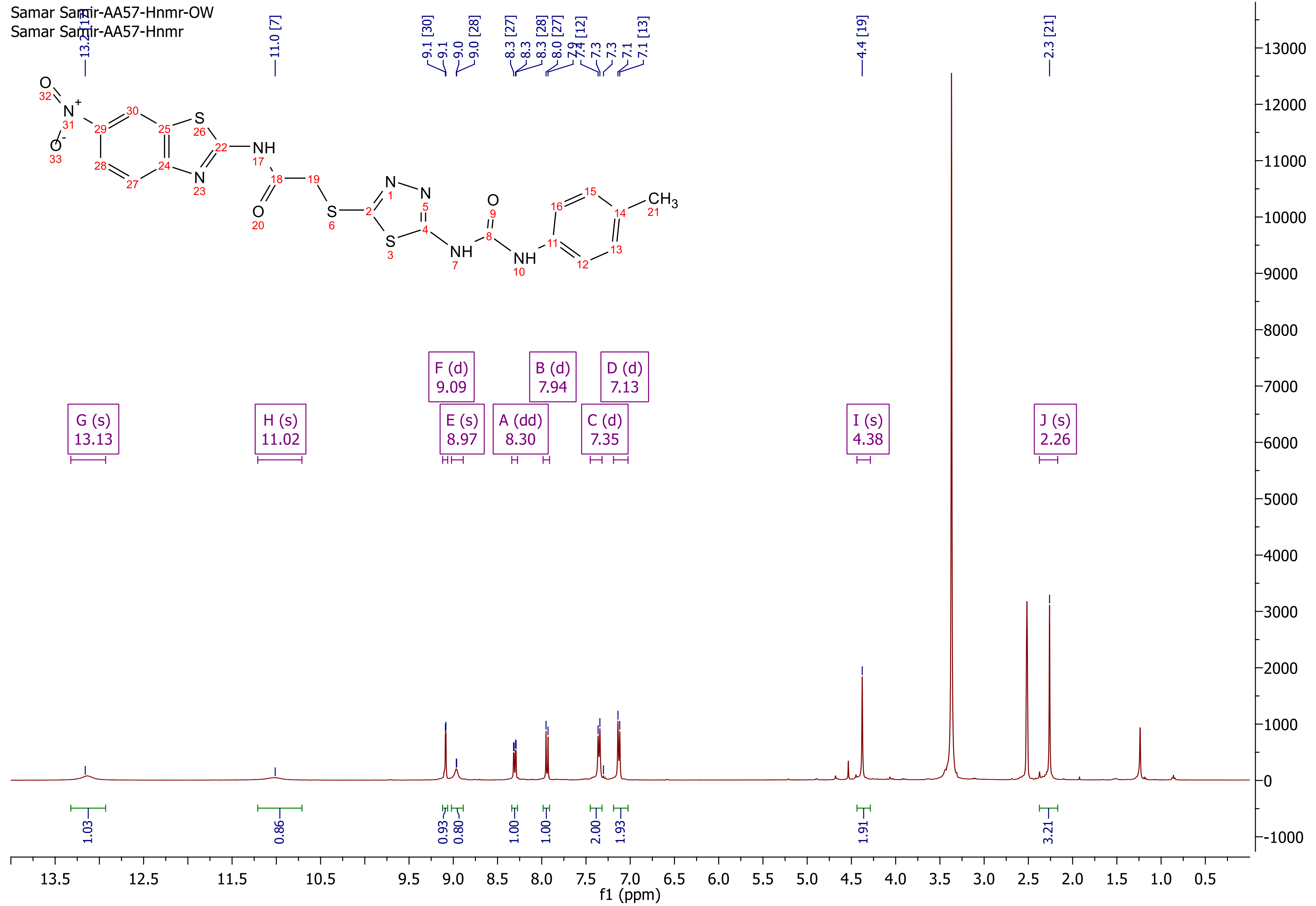

Samar Samir-AA57-Hnmr-OW
Samar Samir-AA57-Hnmr
13.2 [17]
11.0 [7]
9.1 [30]
9.1
9.0
9.0 [28]
8.3 [27]
8.3
8.3 [28]
8.0 [27]
7.9
7.4 [12]
7.3
7.3
7.1
7.1 [13]
4.4 [19]
2.3 [21]
G (s) 13.13
H (s) 11.02
F (d) 9.09
E (s) 8.97
A (dd) 8.30
B (d) 7.94
C (d) 7.35
D (d) 7.13
I (s) 4.38
J (s) 2.26
1.03
0.86
0.93
0.80
1.00
1.00
2.00
1.93
1.91
3.21
f1 (ppm)

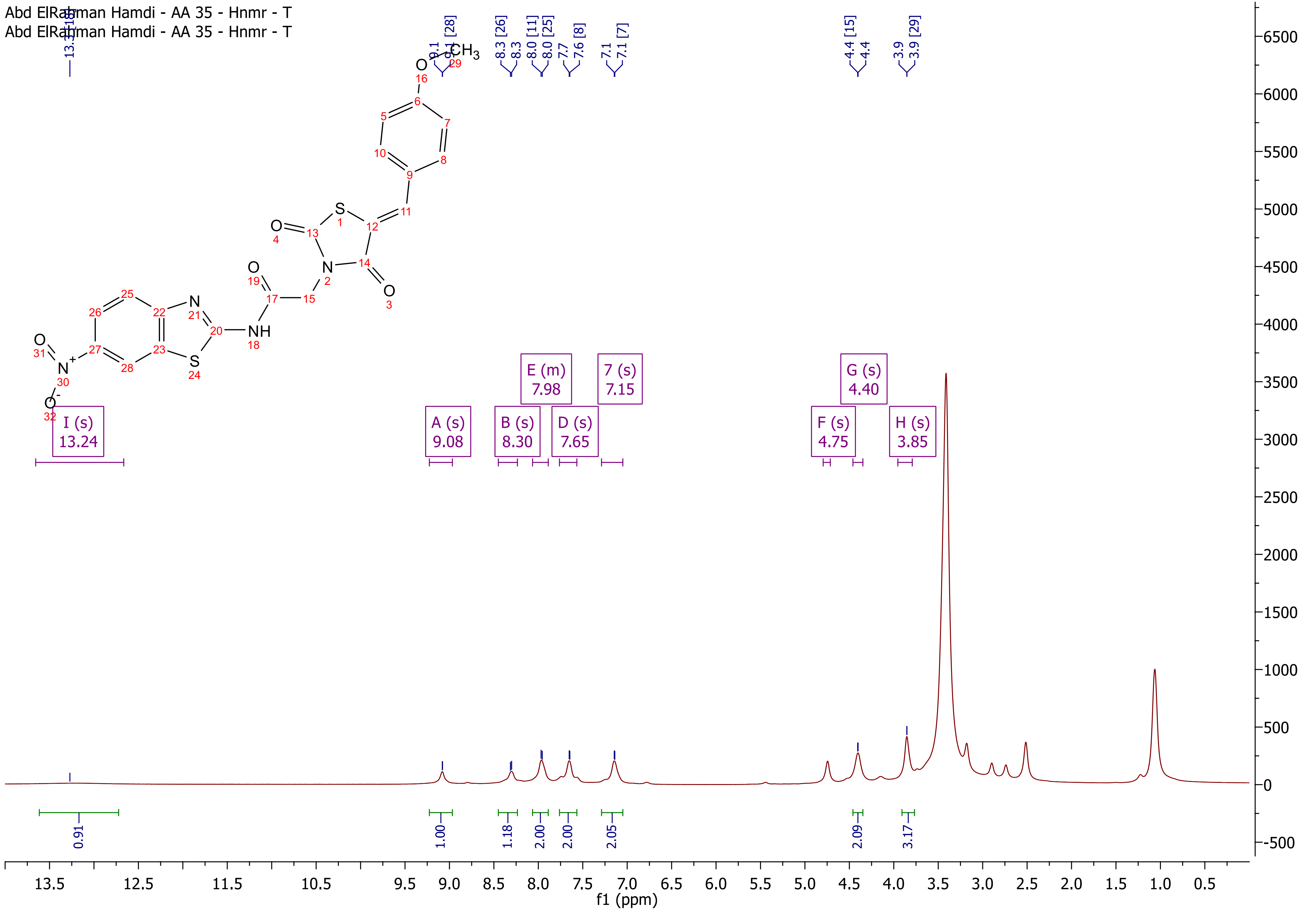

Abd ElRahman Hamdi - AA 35 - Hnmr - T
Abd ElRahman Hamdi - AA 35 - Hnmr - T
I (s)
13.24
A (s)
9.08
B (s)
8.30
E (m)
7.98
D (s)
7.65
7 (s)
7.15
F (s)
4.75
G (s)
4.40
H (s)
3.85
0.91
1.00
1.18
2.00
2.00
2.05
2.09
3.17
f1 (ppm)

Samar Samir-AA30-Hnmr-OW
Samar Samir-AA30-Hnmr

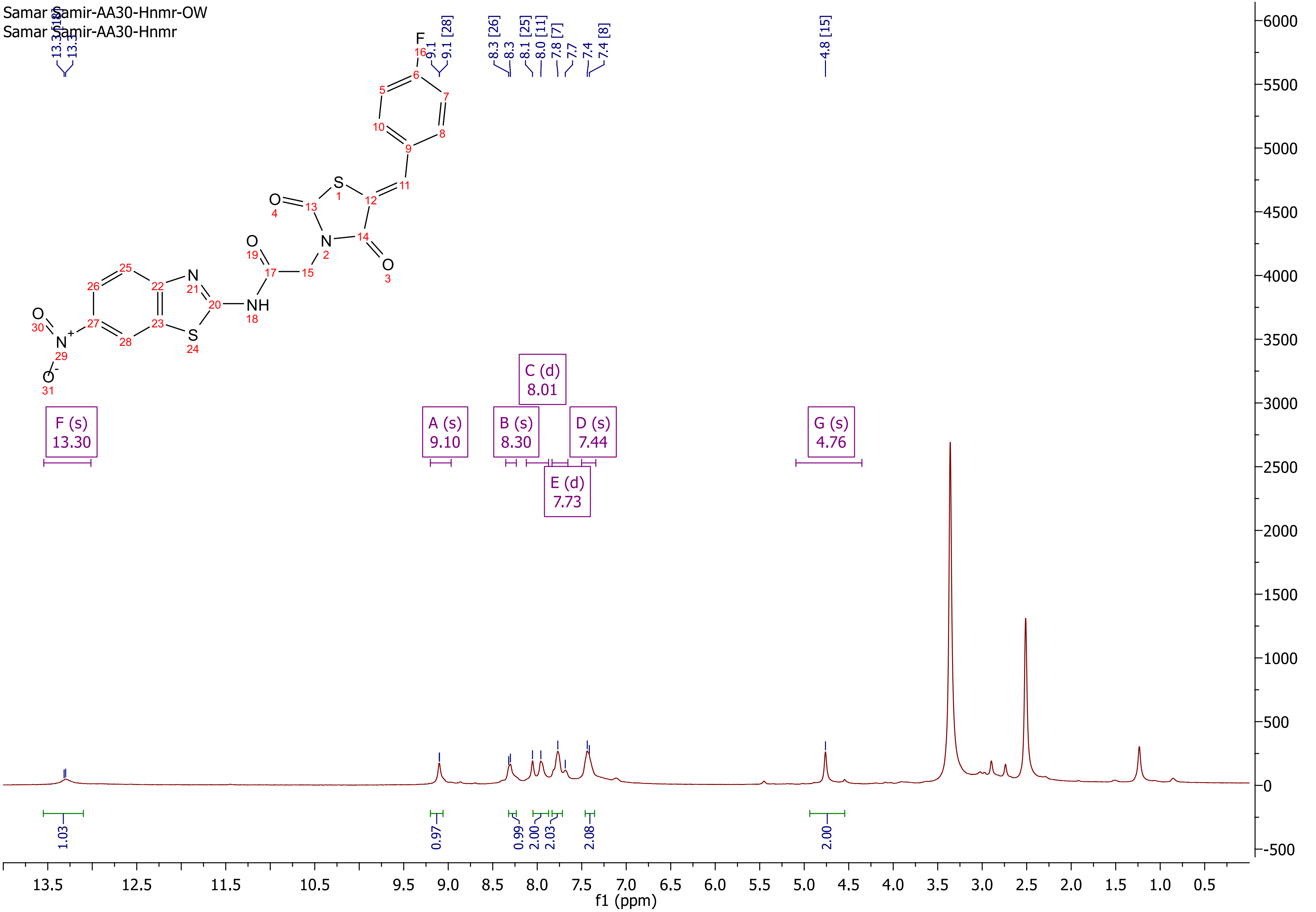

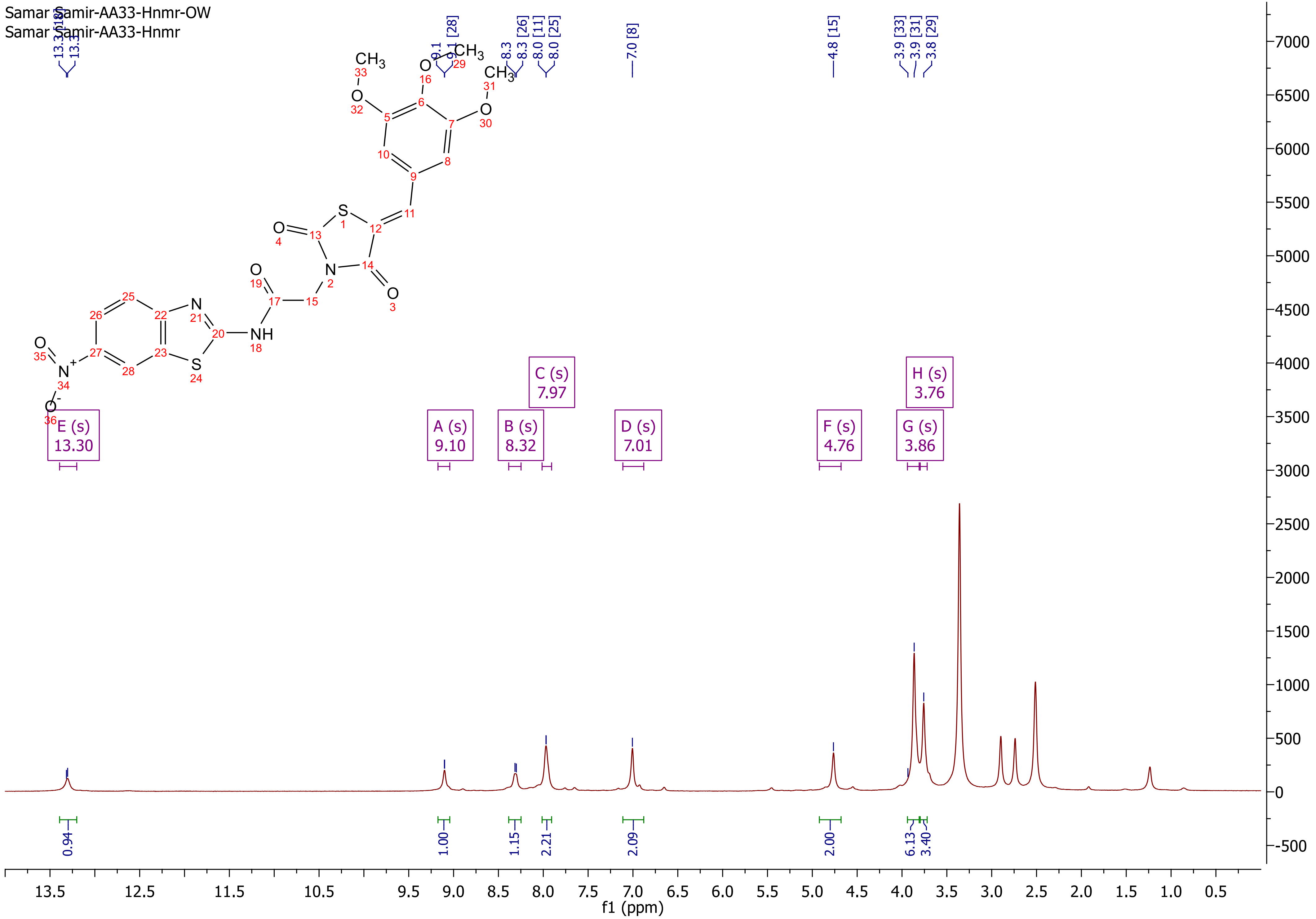
Samar Samir-AA33-Hnmr-OW
Samar Samir-AA33-Hnmr
E (s) 13.30
A (s) 9.10
B (s) 8.32
C (s) 7.97
D (s) 7.01
F (s) 4.76
G (s) 3.86
H (s) 3.76
0.94
1.00
1.15
2.21
2.09
2.00
6.13
3.40
f1 (ppm)

Abd ElRahman Hamdi - AA 50 - Hnmr - T
Abd ElRahman Hamdi - AA 50 - Hnmr - T

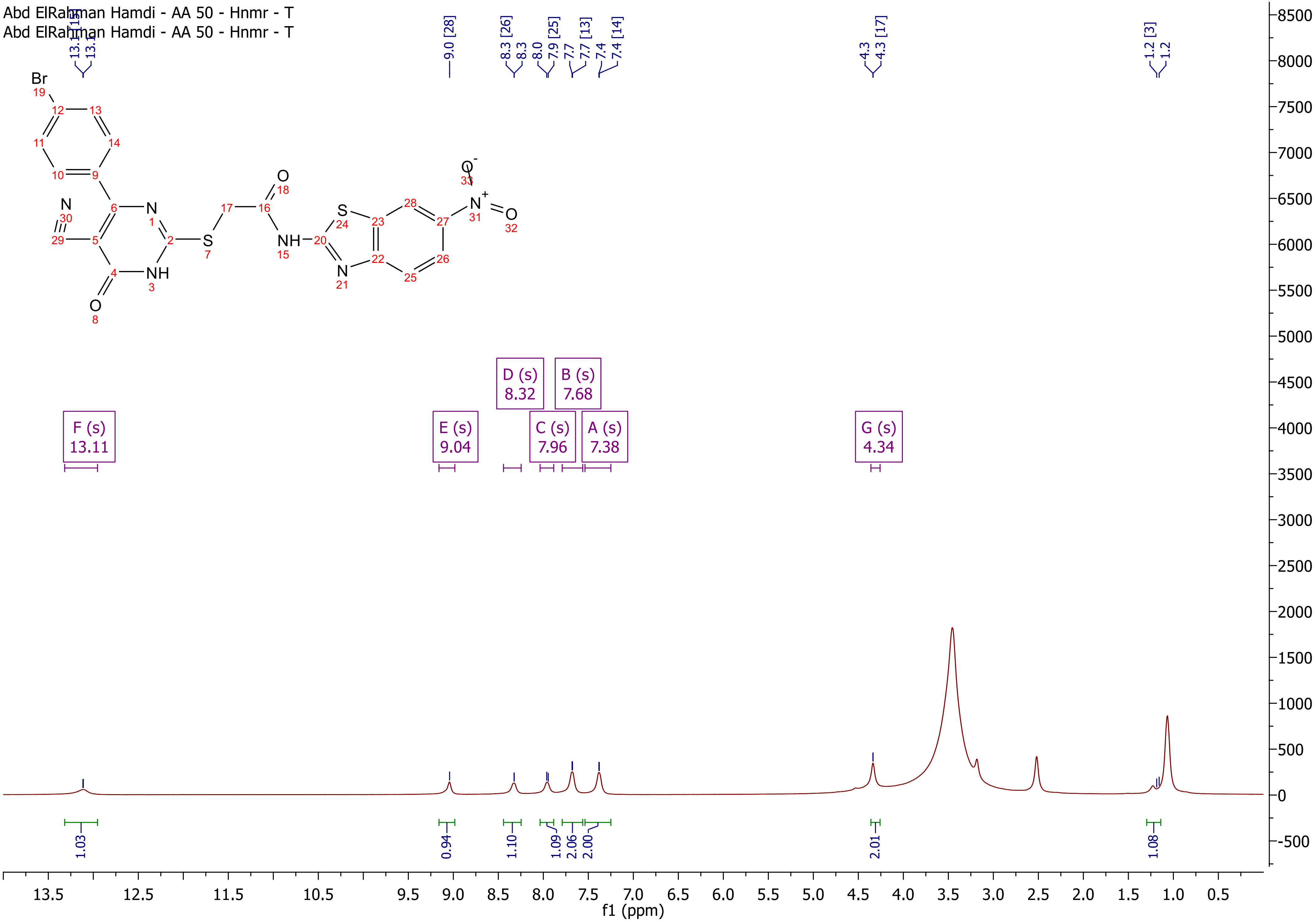

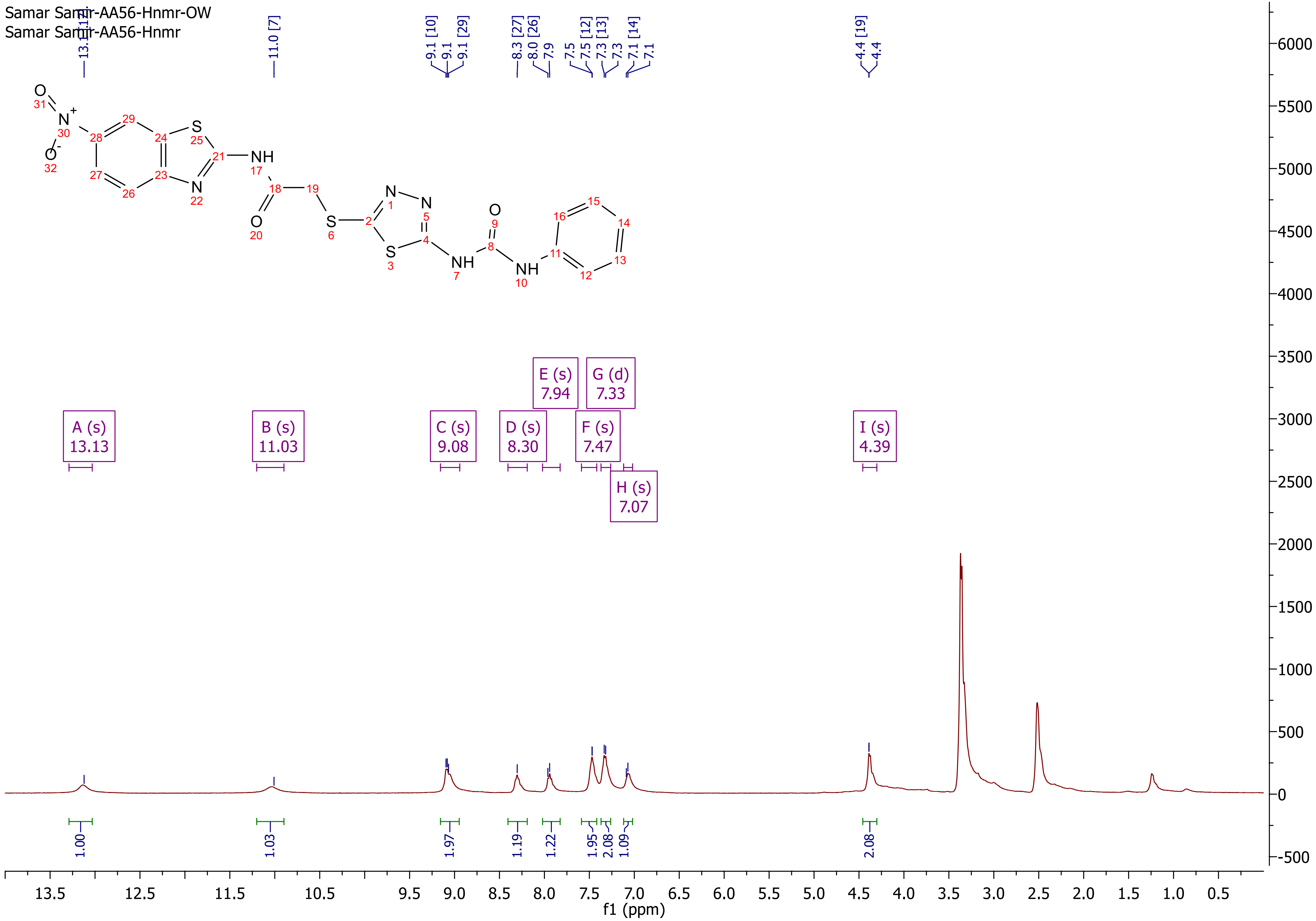

Samar Samir-AA56-Hnmr-OW
Samar Samir-AA56-Hnmr
A (s) 13.13
B (s) 11.03
C (s) 9.08
D (s) 8.30
E (s) 7.94
F (s) 7.47
G (d) 7.33
H (s) 7.07
I (s) 4.39
1.00
1.03
1.97
1.19
1.22
1.95
2.08
1.09
2.08
f1 (ppm)

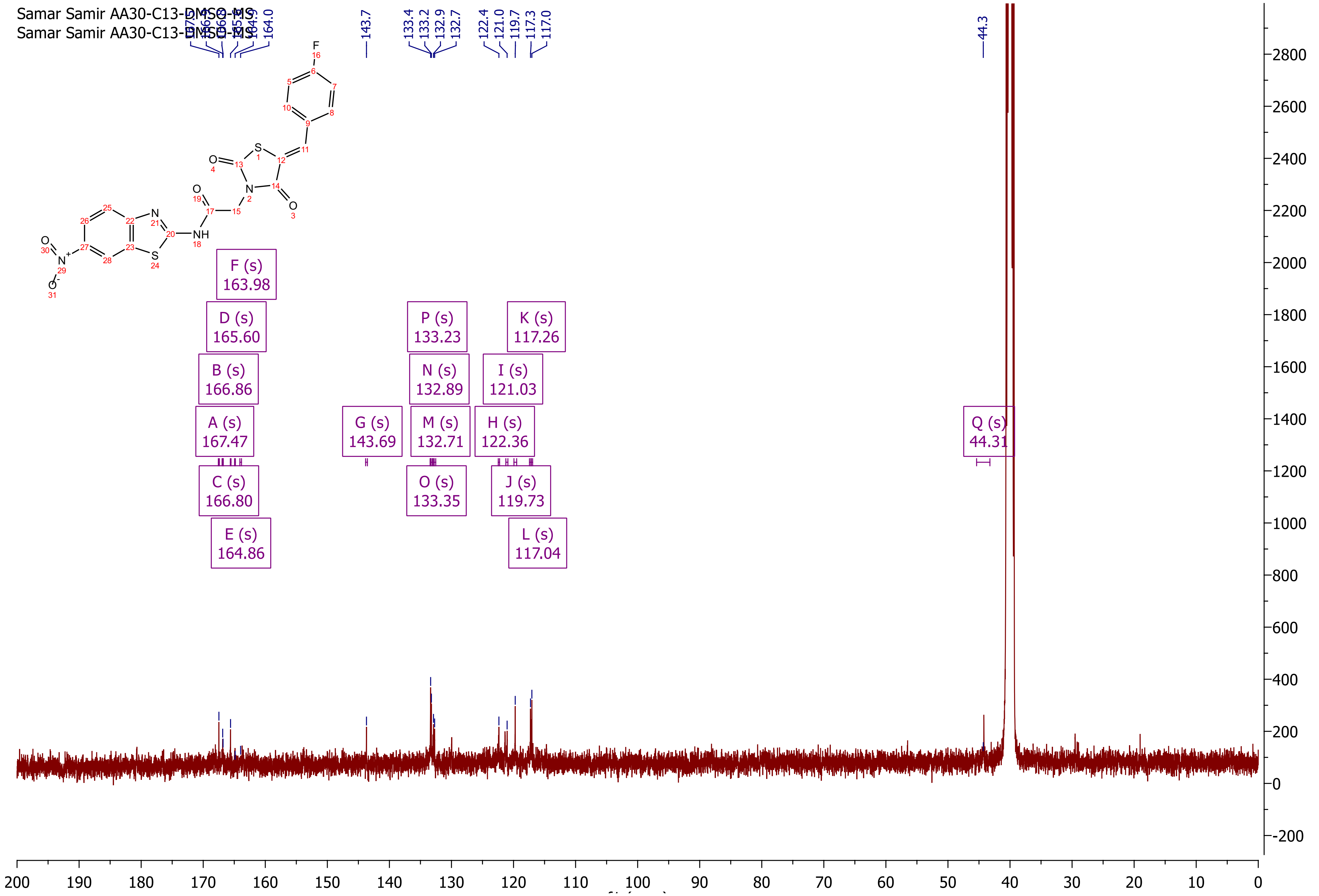
Samar Samir AA30-C13-DMSO-MS
Samar Samir AA30-C13-DMSO-MS
164.9
164.0
143.7
133.4
133.2
132.9
132.7
122.4
121.0
119.7
117.3
117.0
44.3
F (s) 163.98
D (s) 165.60
B (s) 166.86
A (s) 167.47
C (s) 166.80
E (s) 164.86
G (s) 143.69
P (s) 133.23
N (s) 132.89
M (s) 132.71
O (s) 133.35
K (s) 117.26
I (s) 121.03
H (s) 122.36
J (s) 119.73
L (s) 117.04
Q (s) 44.31
2800
2600
2400
2200
2000
1800
1600
1400
1200
1000
800
600
400
200
0
-200
200
190
180
170
160
150
140
130
120
110
100
90
80
70
60
50
40
30
20
10
0

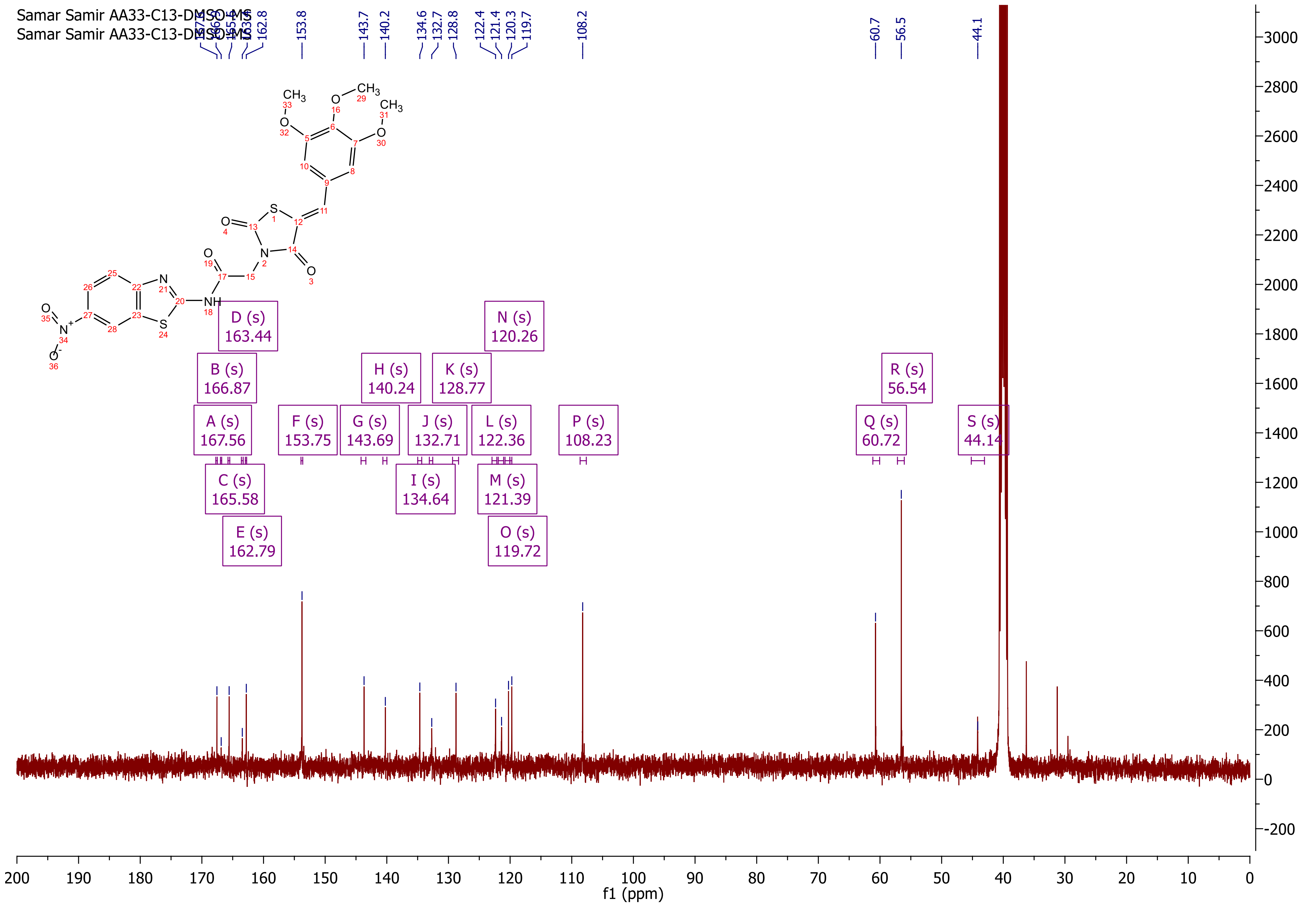

Samar Samir AA33-C13-DMSO-MS
Samar Samir AA33-C13-DMSO-MS
162.8
153.8
143.7
140.2
134.6
132.7
128.8
122.4
121.4
120.3
119.7
108.2
60.7
56.5
44.1
A (s) 167.56
B (s) 166.87
C (s) 165.58
D (s) 163.44
E (s) 162.79
F (s) 153.75
G (s) 143.69
H (s) 140.24
I (s) 134.64
J (s) 132.71
K (s) 128.77
L (s) 122.36
M (s) 121.39
N (s) 120.26
O (s) 119.72
P (s) 108.23
Q (s) 60.72
R (s) 56.54
S (s) 44.14
f1 (ppm)

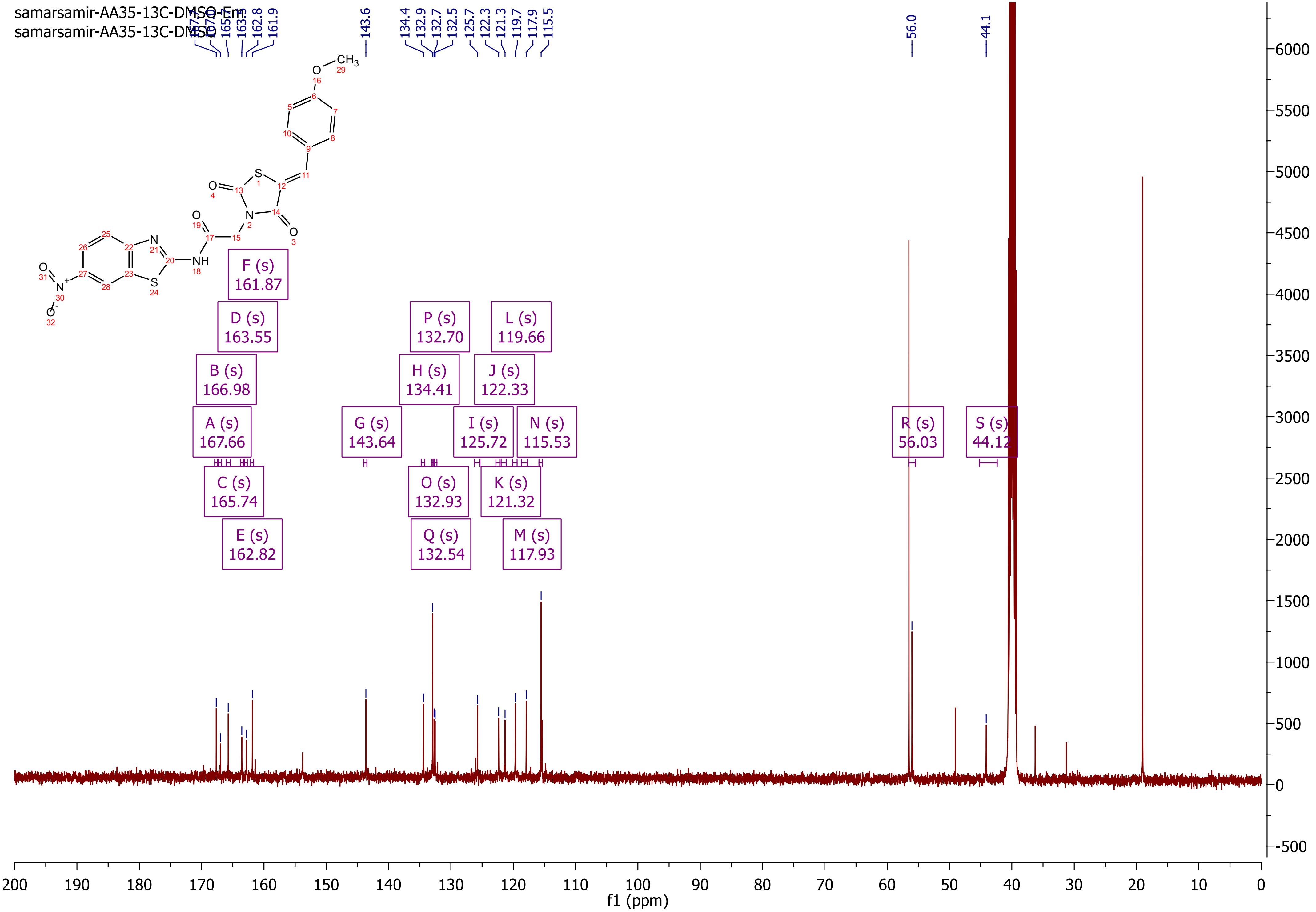
samarsamir-AA35-13C-DMSO-Em
samarsamir-AA35-13C-DMSO
162.8
161.9
143.6
134.4
132.9
132.7
132.5
125.7
122.3
121.3
119.7
117.9
115.5
56.0
44.1
A (s) 167.66
B (s) 166.98
C (s) 165.74
D (s) 163.55
E (s) 162.82
F (s) 161.87
G (s) 143.64
H (s) 134.41
I (s) 125.72
J (s) 122.33
K (s) 121.32
L (s) 119.66
M (s) 117.93
N (s) 115.53
O (s) 132.93
P (s) 132.70
Q (s) 132.54
R (s) 56.03
S (s) 44.12
f1 (ppm)

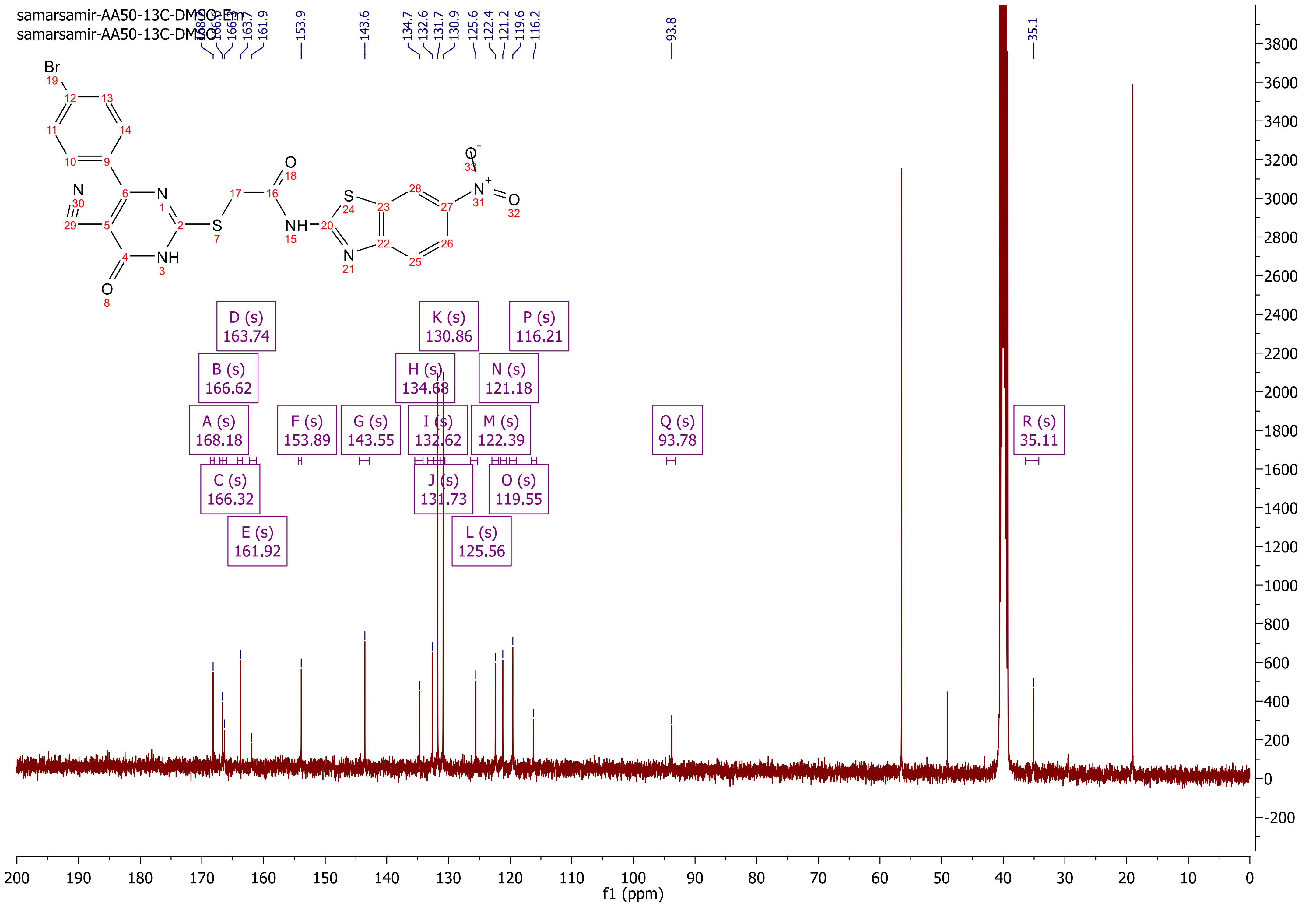
samarsamir-AA50-13C-DMSO-Em
samarsamir-AA50-13C-DMSO
163.7
161.9
153.9
143.6
134.7
132.6
131.7
130.9
125.6
122.4
121.2
119.6
116.2
93.8
35.1
Br
N
N
NH
O
S
O
NH
S
N
N
O
O
A (s) 168.18
B (s) 166.62
C (s) 166.32
D (s) 163.74
E (s) 161.92
F (s) 153.89
G (s) 143.55
H (s) 134.68
I (s) 132.62
J (s) 131.73
K (s) 130.86
L (s) 125.56
M (s) 122.39
N (s) 121.18
O (s) 119.55
P (s) 116.21
Q (s) 93.78
R (s) 35.11
f1 (ppm)

Samar Samir AA51-C13-DMSO-MS
Samar Samir AA51-C13-DMSO-MS

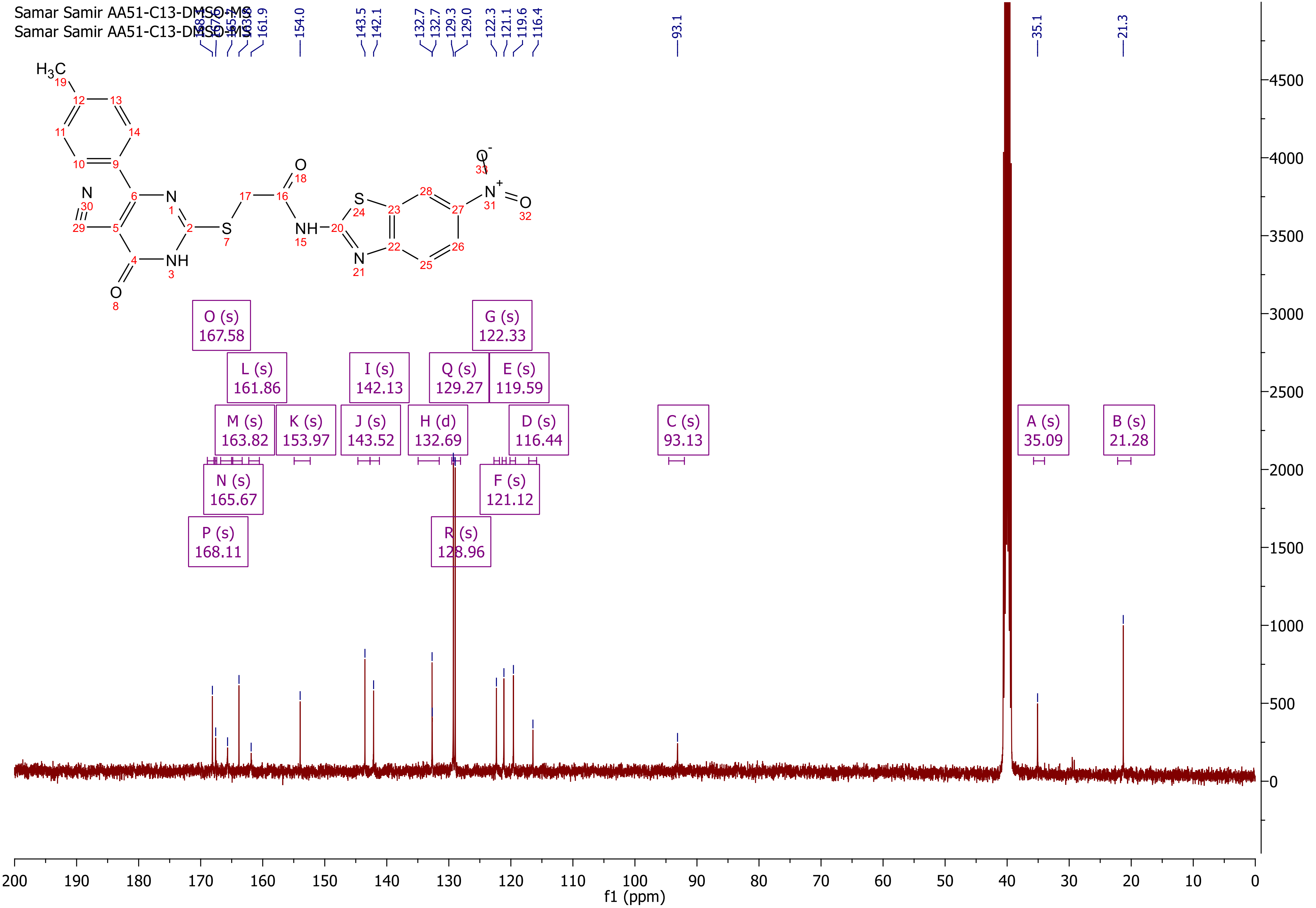

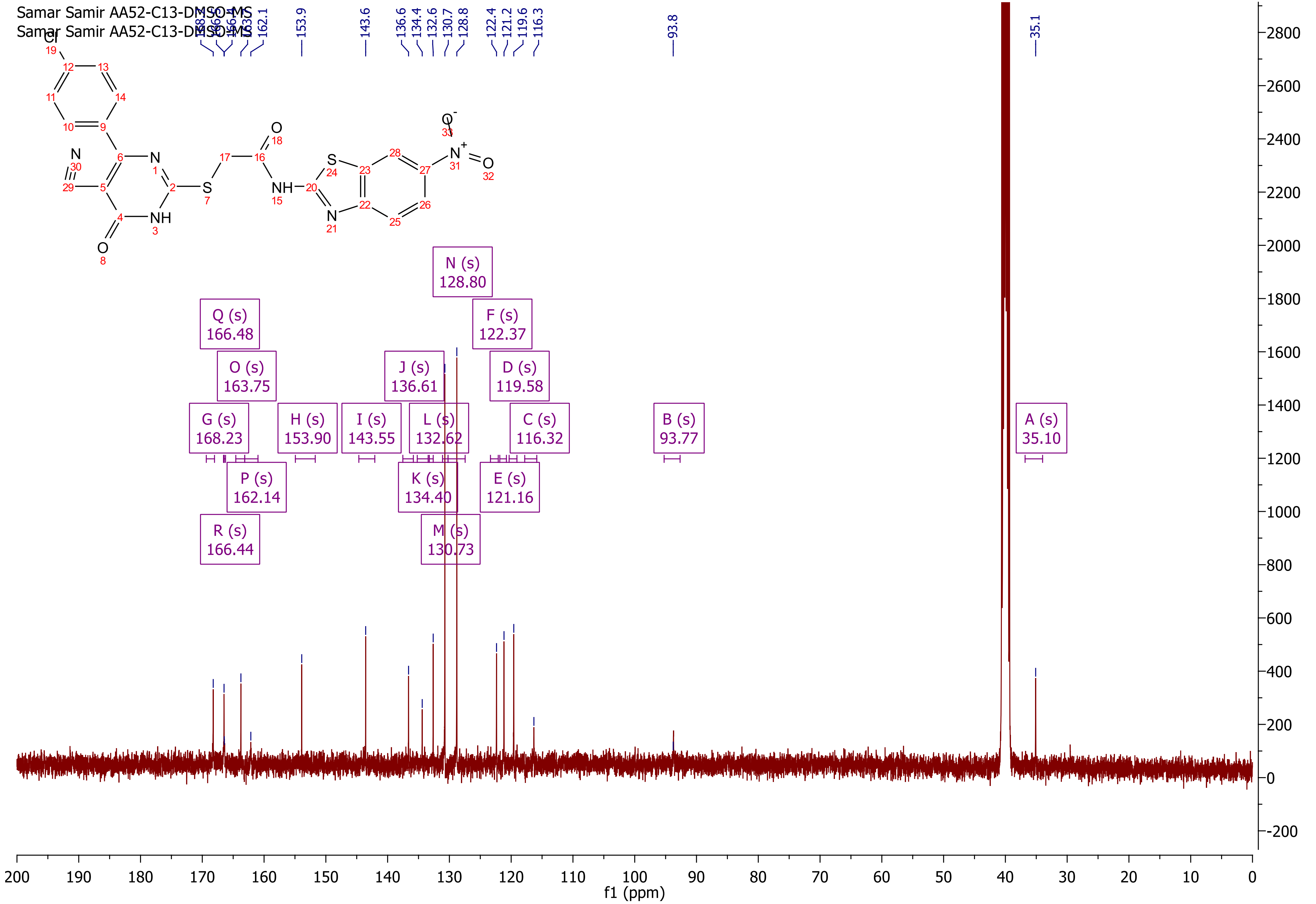

Samar Samir AA52-C13-DMSO-MS
Samar Samir AA52-C13-DMSO-MS
153.9
143.6
136.6
134.4
132.6
130.7
128.8
122.4
121.2
119.6
116.3
93.8
35.1
N (s) 128.80
Q (s) 166.48
F (s) 122.37
O (s) 163.75
J (s) 136.61
D (s) 119.58
G (s) 168.23
H (s) 153.90
I (s) 143.55
L (s) 132.62
C (s) 116.32
B (s) 93.77
A (s) 35.10
P (s) 162.14
K (s) 134.40
E (s) 121.16
R (s) 166.44
M (s) 130.73
f1 (ppm)

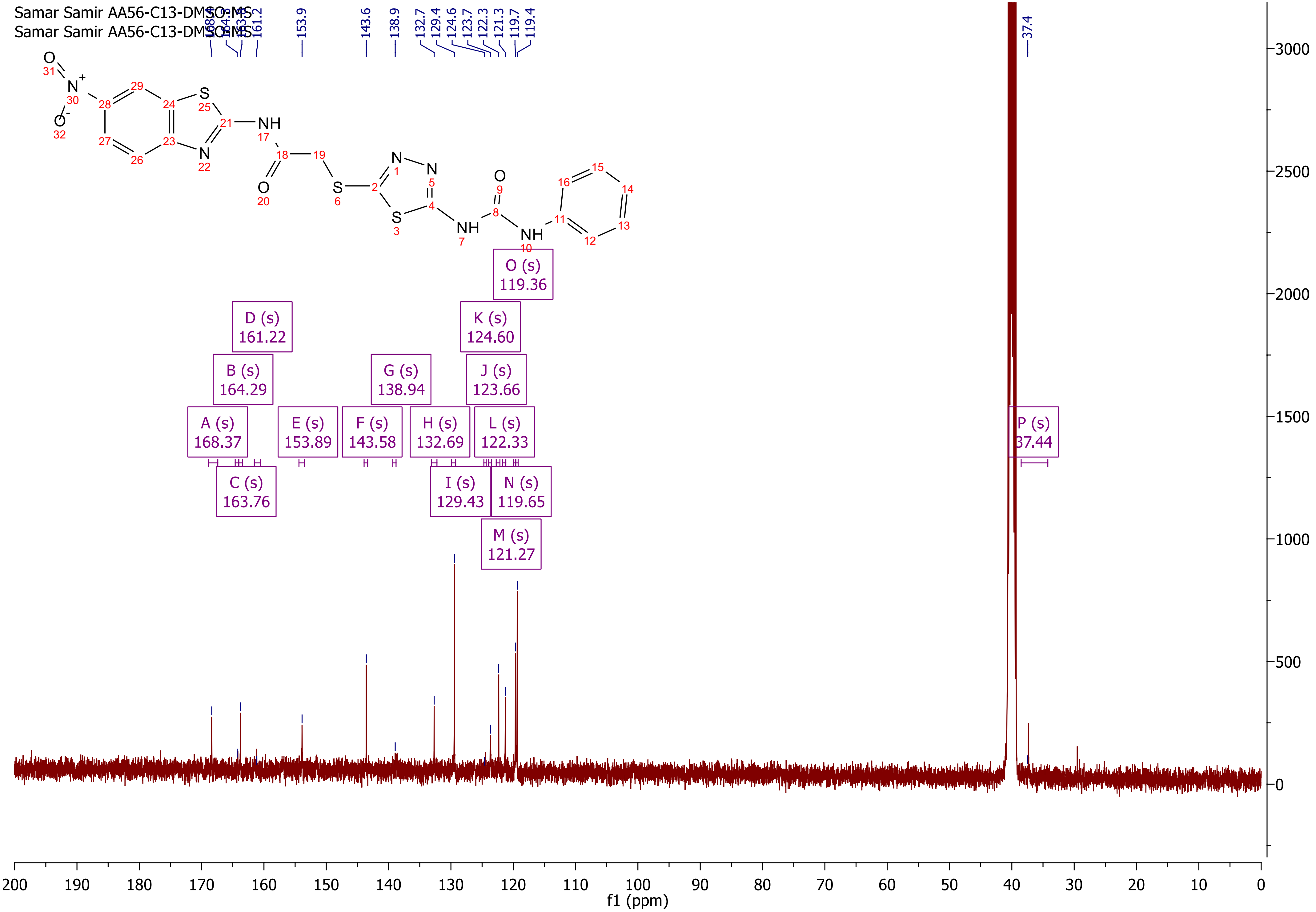

Samar Samir AA56-C13-DMSO-MS
Samar Samir AA56-C13-DMSO-MS
161.2
153.9
143.6
138.9
132.7
129.4
124.6
123.7
122.3
121.3
119.7
119.4
37.4
A (s) 168.37
B (s) 164.29
C (s) 163.76
D (s) 161.22
E (s) 153.89
F (s) 143.58
G (s) 138.94
H (s) 132.69
I (s) 129.43
J (s) 123.66
K (s) 124.60
L (s) 122.33
M (s) 121.27
N (s) 119.65
O (s) 119.36
P (s) 37.44
f1 (ppm)

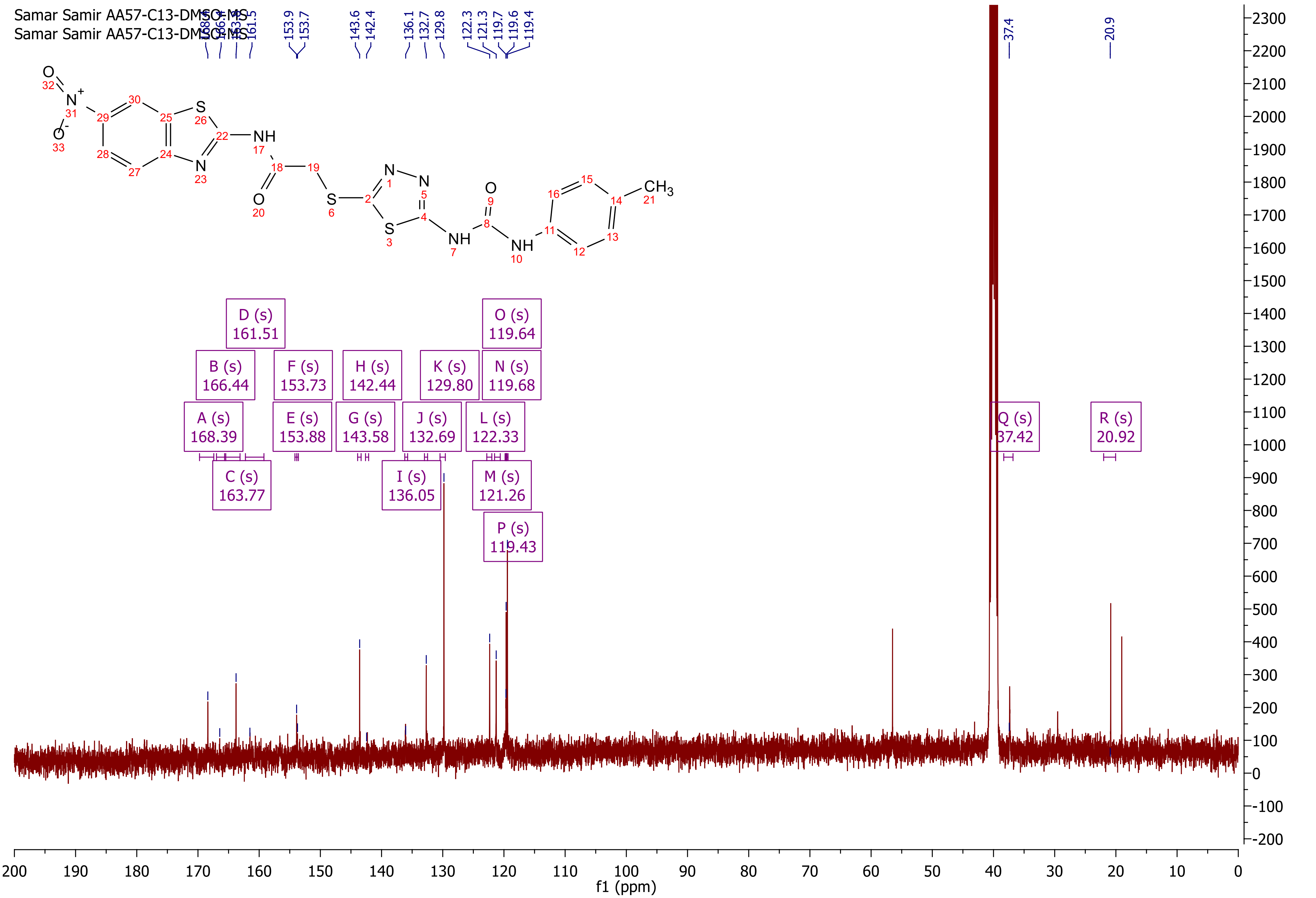

Samar Samir AA57-C13-DMSO-MS
Samar Samir AA57-C13-DMSO-MS
153.9
153.7
143.6
142.4
136.1
132.7
129.8
122.3
121.3
119.7
119.6
119.4
37.4
20.9
A (s) 168.39
B (s) 166.44
C (s) 163.77
D (s) 161.51
E (s) 153.88
F (s) 153.73
G (s) 143.58
H (s) 142.44
I (s) 136.05
J (s) 132.69
K (s) 129.80
L (s) 122.33
M (s) 121.26
N (s) 119.68
O (s) 119.64
P (s) 119.43
Q (s) 37.42
R (s) 20.92
f1 (ppm)

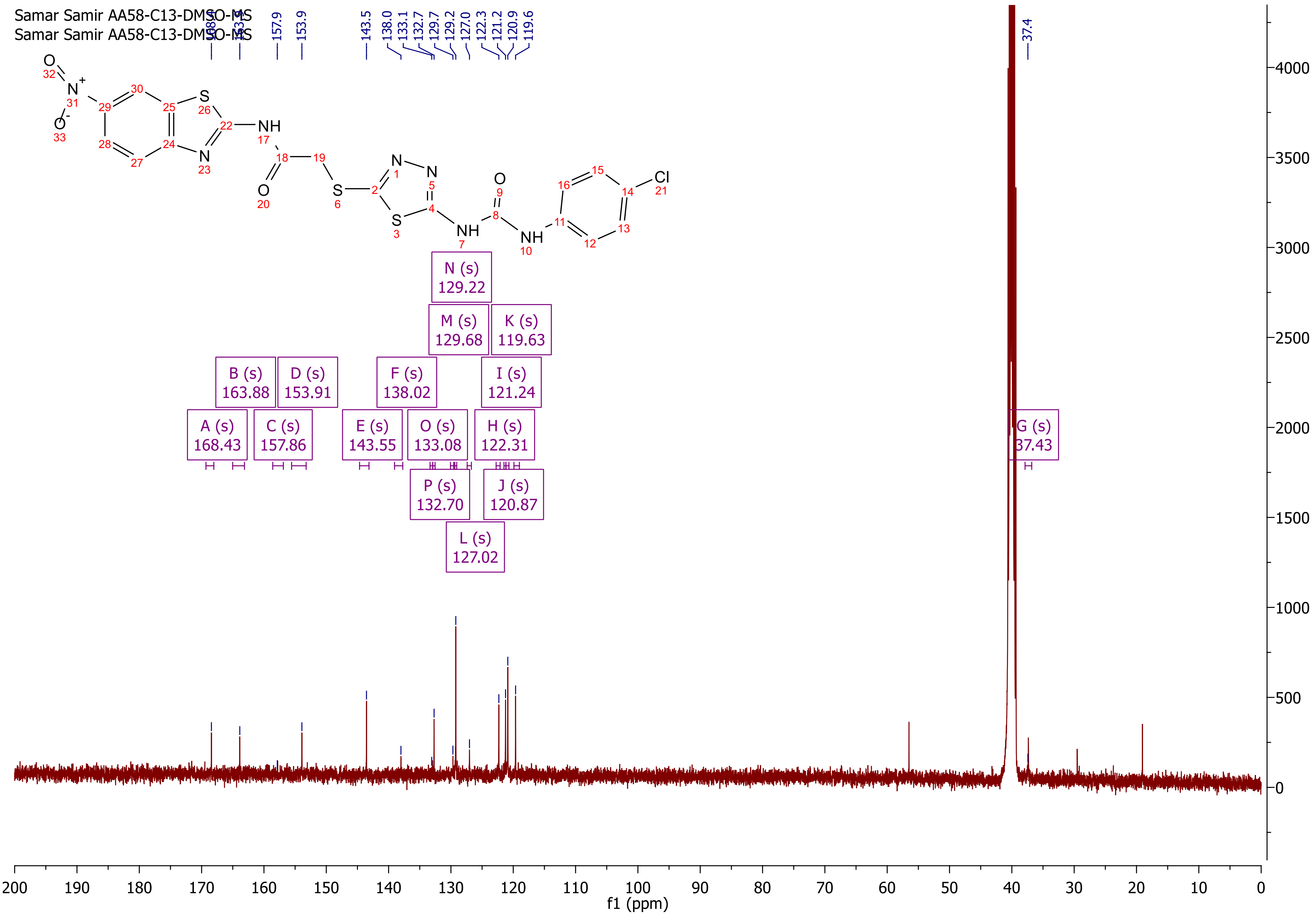
Samar Samir AA58-C13-DMSO-MS
Samar Samir AA58-C13-DMSO-MS
157.9
153.9
143.5
138.0
133.1
132.7
129.7
129.2
127.0
122.3
121.2
120.9
119.6
37.4
N (s) 129.22
M (s) 129.68
K (s) 119.63
B (s) 163.88
D (s) 153.91
F (s) 138.02
I (s) 121.24
A (s) 168.43
C (s) 157.86
E (s) 143.55
O (s) 133.08
H (s) 122.31
G (s) 37.43
P (s) 132.70
J (s) 120.87
L (s) 127.02
f1 (ppm)

Abd El Rahman Hamdy-AH30-Fnmr-A
Abd El Rahman Hamdy-AH30-Fnmr-A

^{19}F NMR (377 MHz, DMSO) δ -108.91 (s).

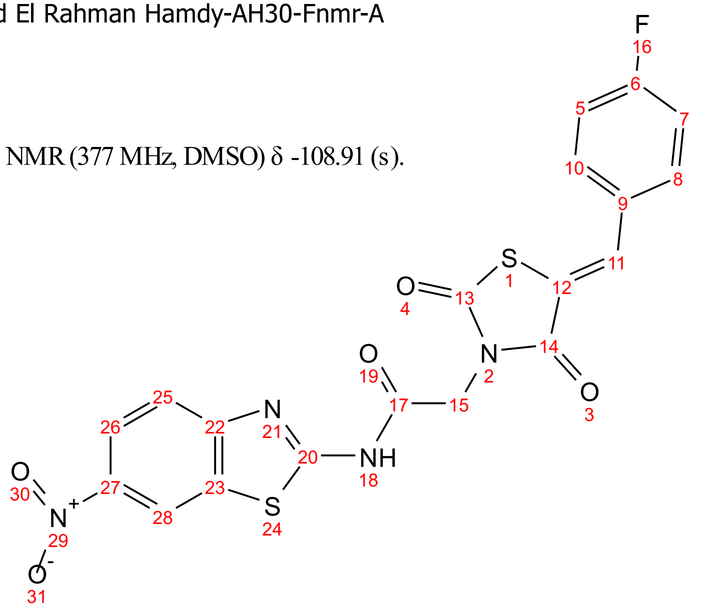

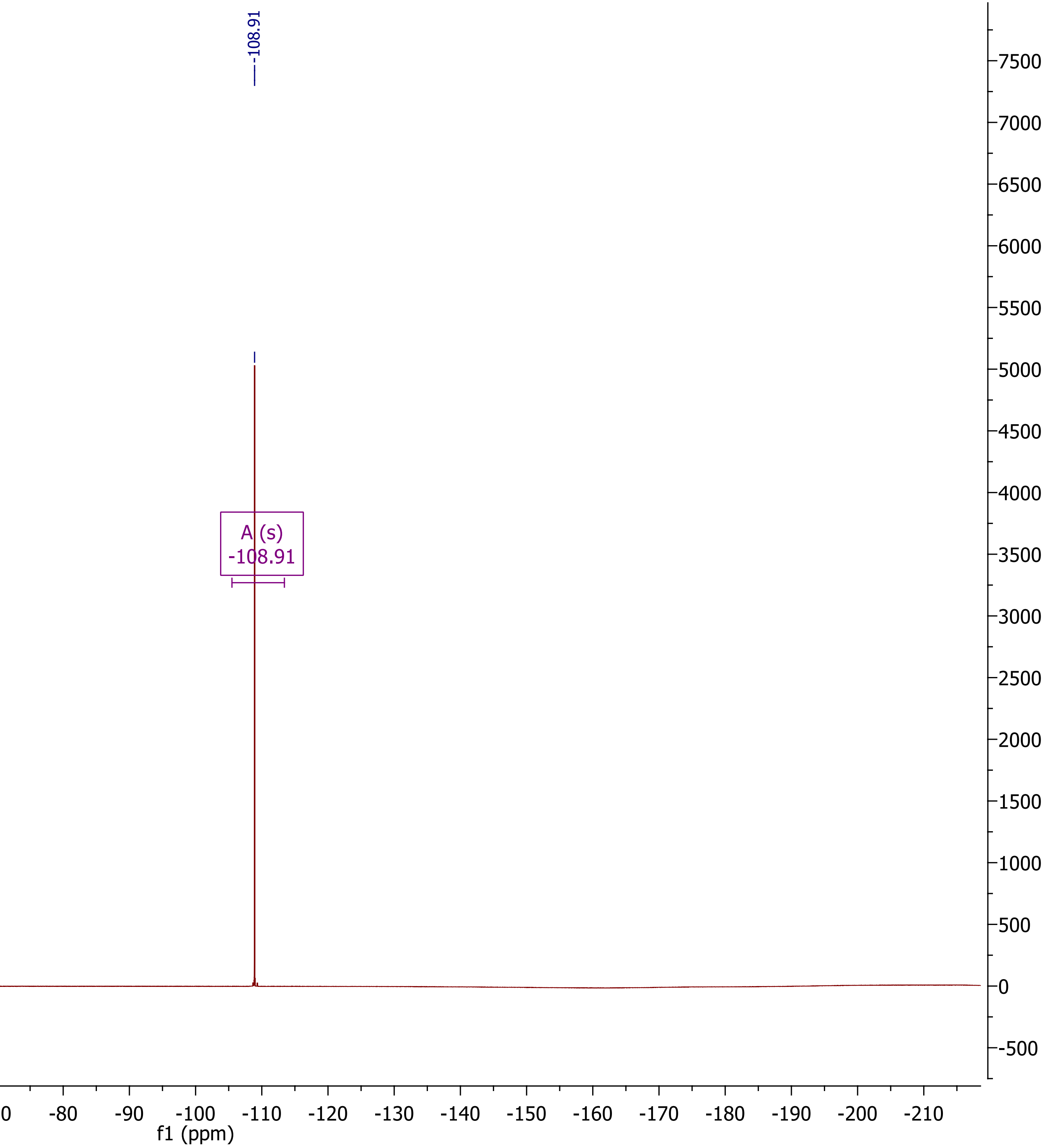

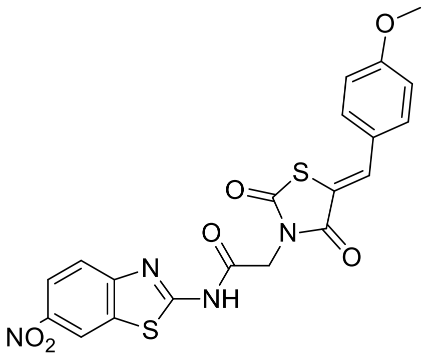

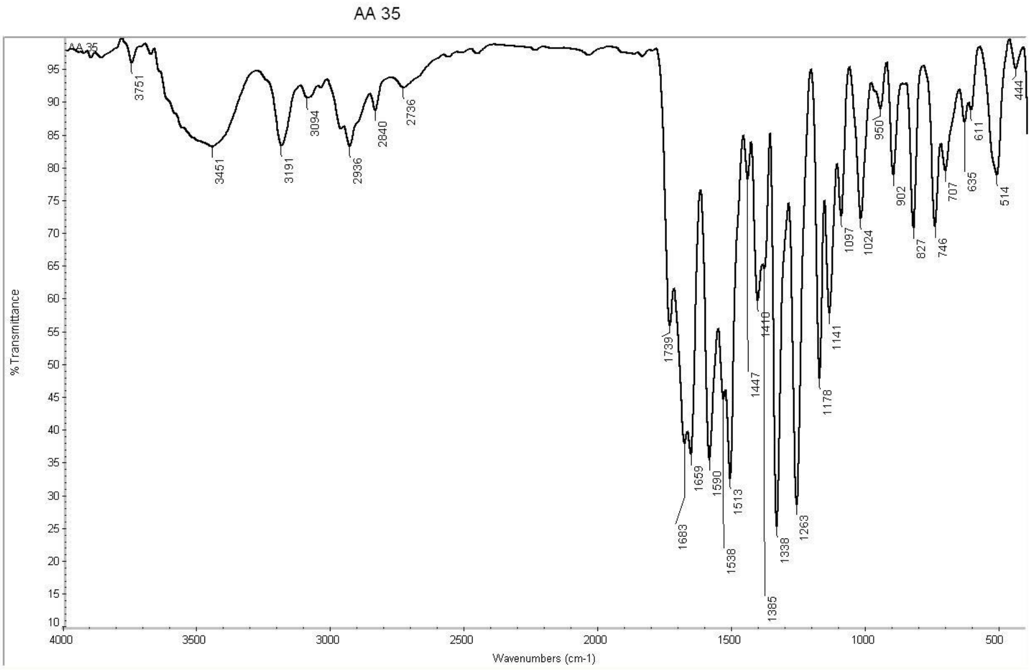
AA 35
% Transmittance
Wavenumbers (cm-1)
3751
3451
3191
3094
2936
2840
2736
1739
1683
1659
1590
1538
1513
1447
1410
1385
1338
1263
1178
1141
1097
1024
950
902
827
746
707
635
611
514
444

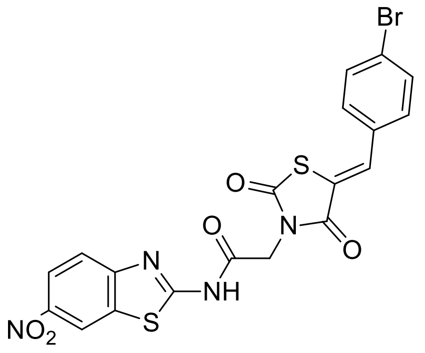
Br
S
O
N
O
O
N
NH
S
NO2

AA 36

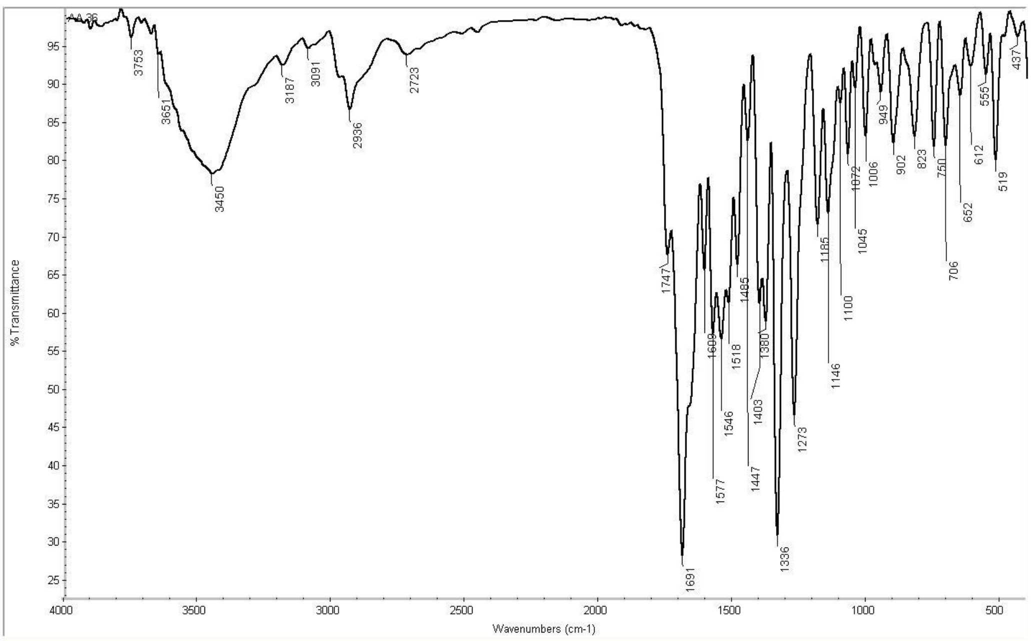
AA 36
% Transmittance
Wavenumbers (cm-1)
4000
3500
3000
2500
2000
1500
1000
500
95
90
85
80
75
70
65
60
55
50
45
40
35
30
25
3753
3651
3450
3187
3091
2936
2723
1747
1691
1609
1577
1546
1518
1485
1447
1403
1380
1336
1273
1185
1146
1100
1072
1045
1006
949
902
823
750
706
652
612
555
519
437

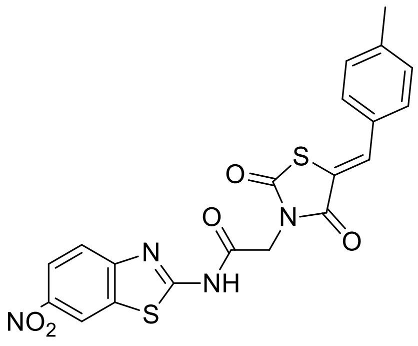
S
O
N
O
O
N
NH
NO2
S

AA 37

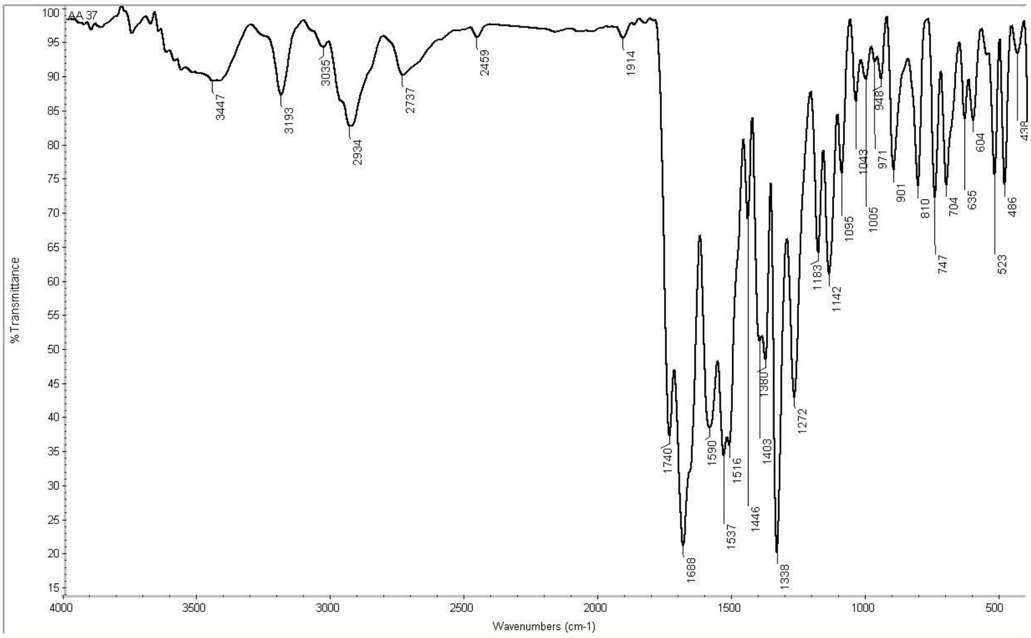
AA 37
%Transmittance
Wavenumbers (cm-1)
3447
3193
3035
2934
2737
2459
1914
1740
1688
1590
1537
1516
1446
1403
1380
1338
1272
1183
1142
1095
1043
1005
971
948
901
810
747
704
635
604
523
486
438

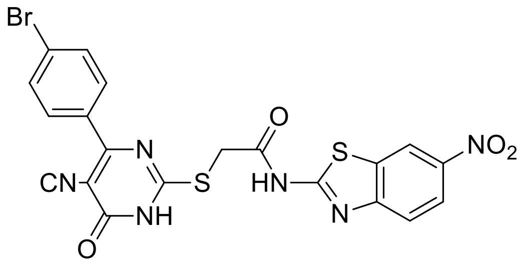
Br
N
CN
NH
O
S
O
HN
S
N
NO2

AA 50

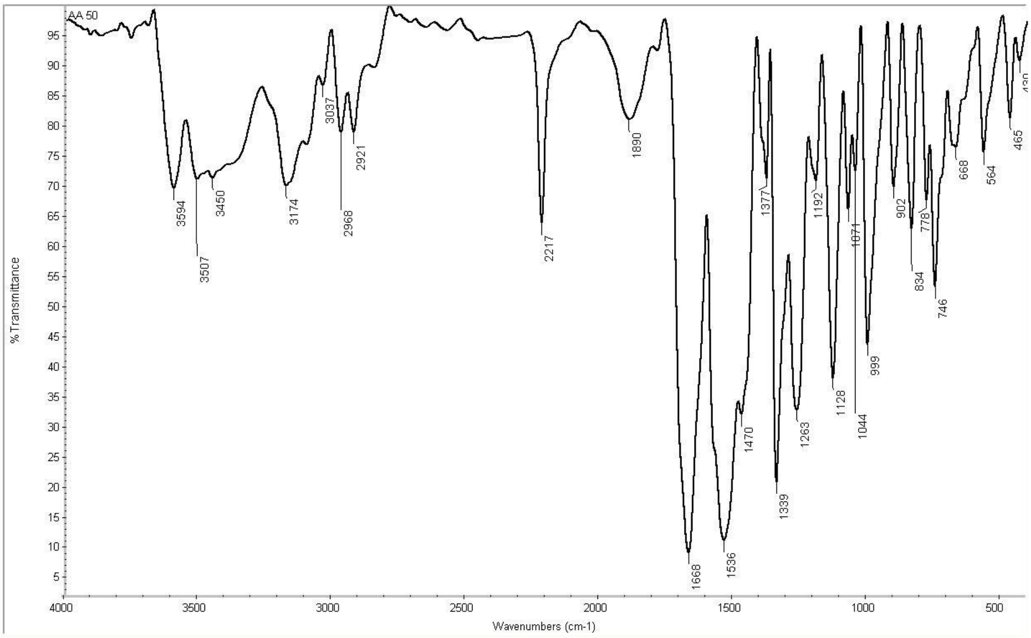
AA 50
% Transmittance
Wavenumbers (cm-1)
3594
3507
3450
3174
3037
2968
2921
2217
1890
1668
1536
1470
1377
1339
1263
1192
1128
1071
1044
999
902
834
778
746
668
564
465

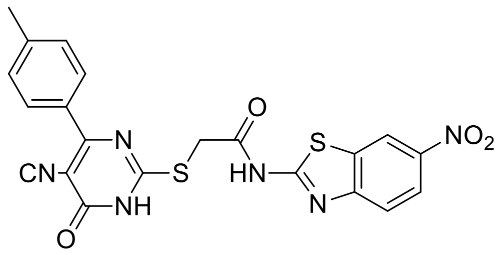
CN
N
NH
O
S
O
HN
S
N
NO2

AA 51

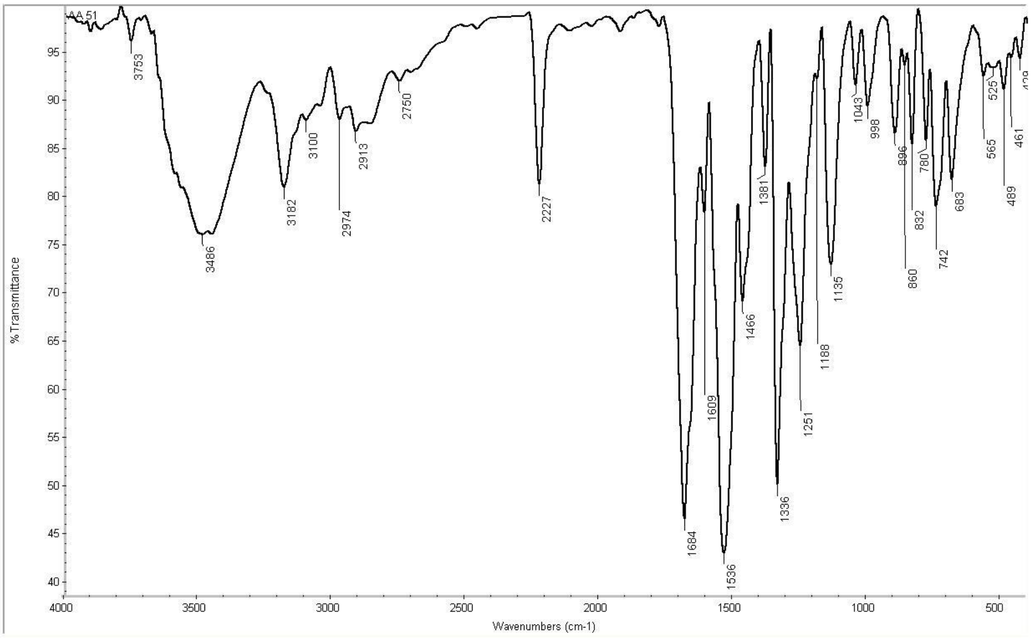
AA 51
% Transmittance
Wavenumbers (cm-1)
3753
3486
3182
3100
2974
2913
2750
2227
1684
1609
1536
1466
1381
1336
1251
1188
1135
1043
998
896
860
832
780
742
683
565
526
489
461

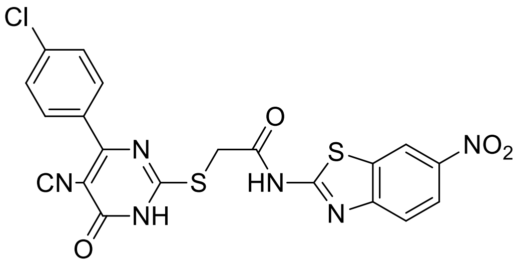

AA 52

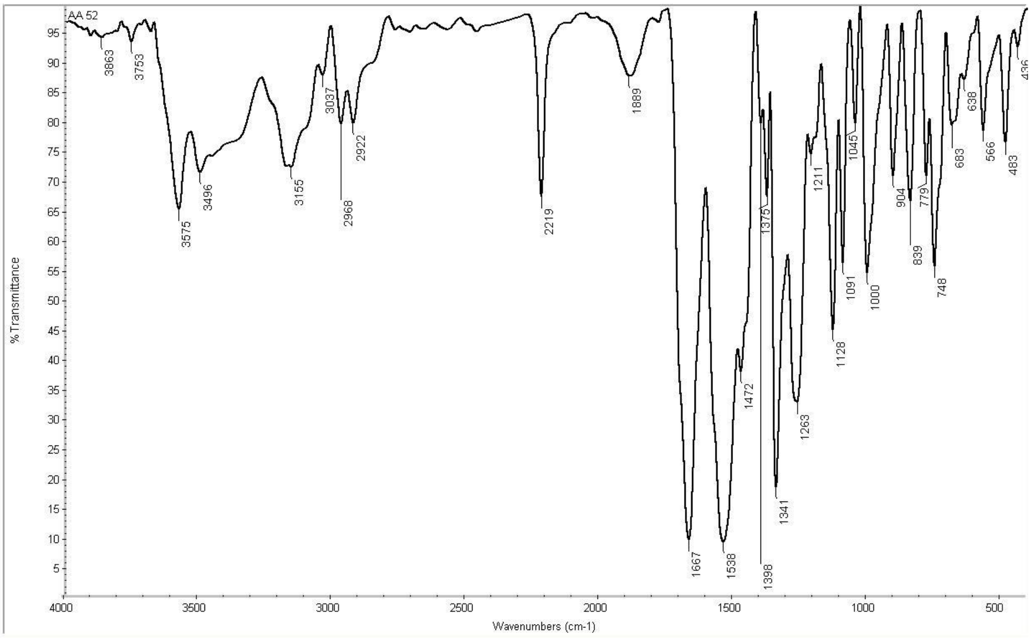

AA 52
% Transmittance
Wavenumbers (cm-1)
3863
3753
3575
3496
3155
3037
2968
2922
2219
1889
1667
1538
1472
1398
1375
1341
1263
1211
1128
1091
1045
1000
904
839
779
748
683
638
566
483
436

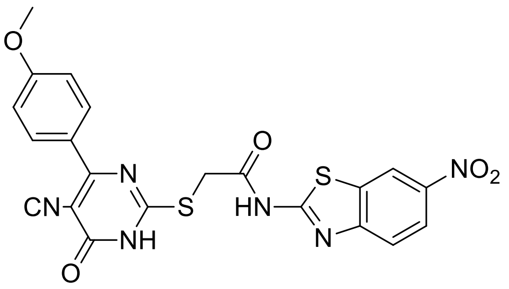

CN
NH
N
S
O
HN
S
N
NO2

AA 53

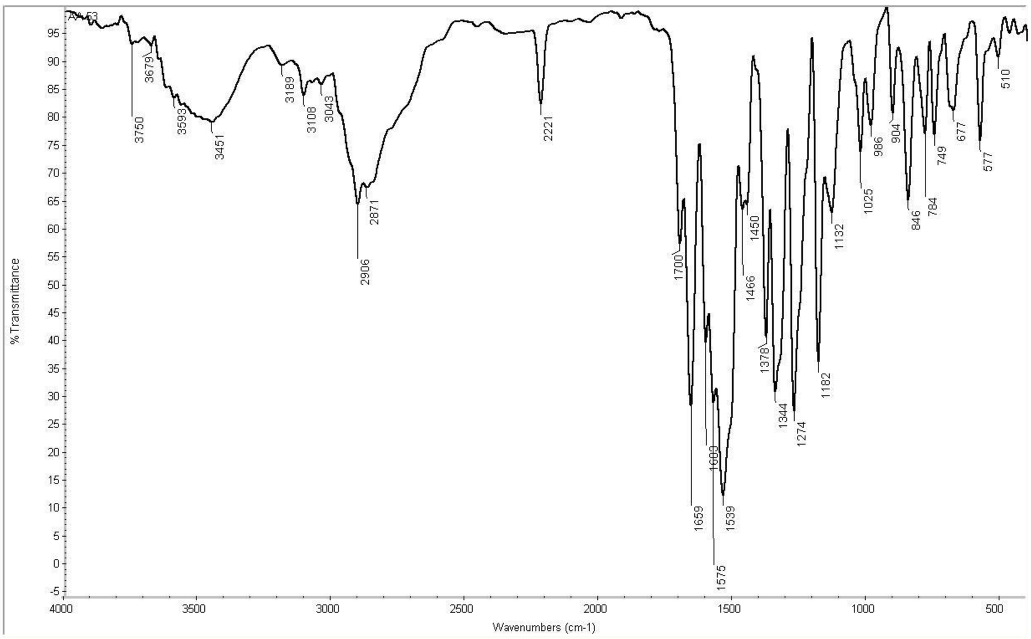

% Transmittance
Wavenumbers (cm-1)
3750
3679
3593
3451
3189
3108
3043
2906
2871
2221
1700
1659
1603
1575
1539
1466
1450
1378
1344
1274
1182
1132
1025
986
904
846
784
749
677
577
510

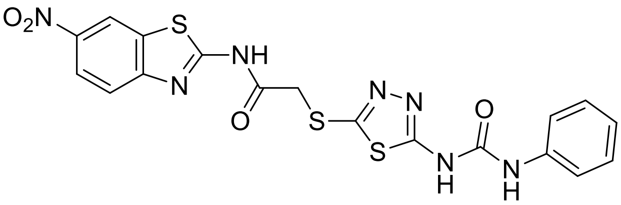

AA 56

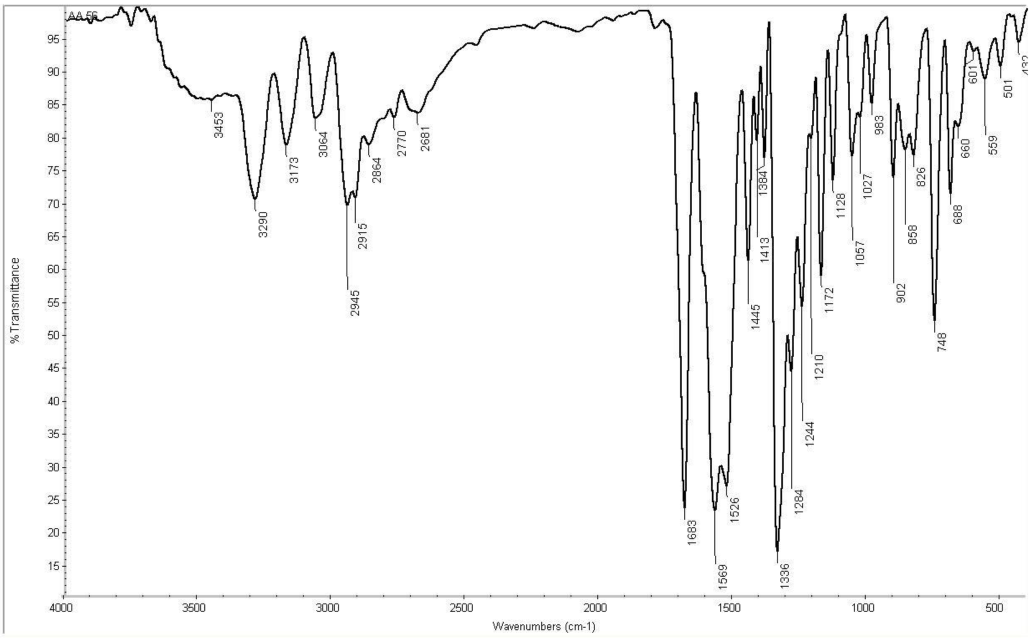
AA 56
%Transmittance
Wavenumbers (cm-1)
3453
3290
3173
3064
2945
2915
2864
2770
2681
1683
1569
1526
1445
1413
1384
1336
1284
1244
1210
1172
1128
1057
1027
983
902
858
826
748
688
660
601
559
501

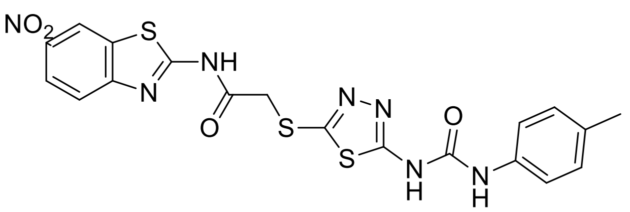
NO2
S
NH
N
O
S
N
N
S
O
N
H
N
H

AA 57

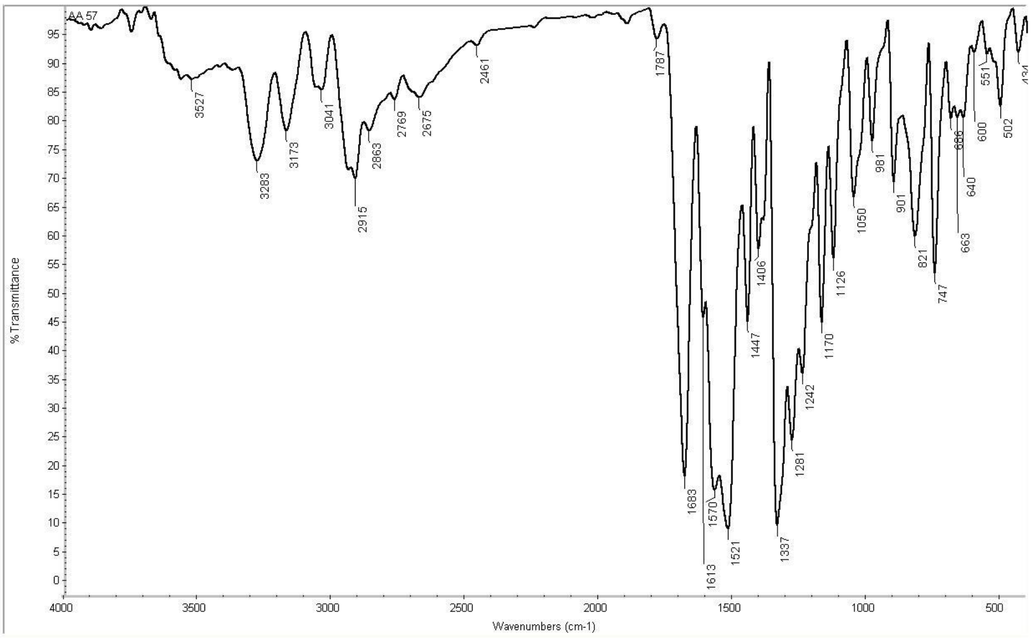
AA 57
% Transmittance
Wavenumbers (cm-1)
3527
3283
3173
3041
2915
2863
2769
2675
2461
1787
1683
1613
1570
1521
1447
1406
1337
1281
1242
1170
1126
1050
981
901
821
747
686
663
640
600
551
502

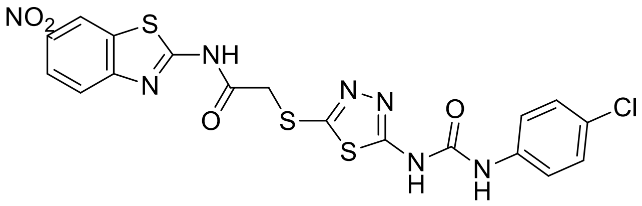
NO2
S
NH
N
O
S
N
N
S
O
N
H
N
H
Cl

AA 58

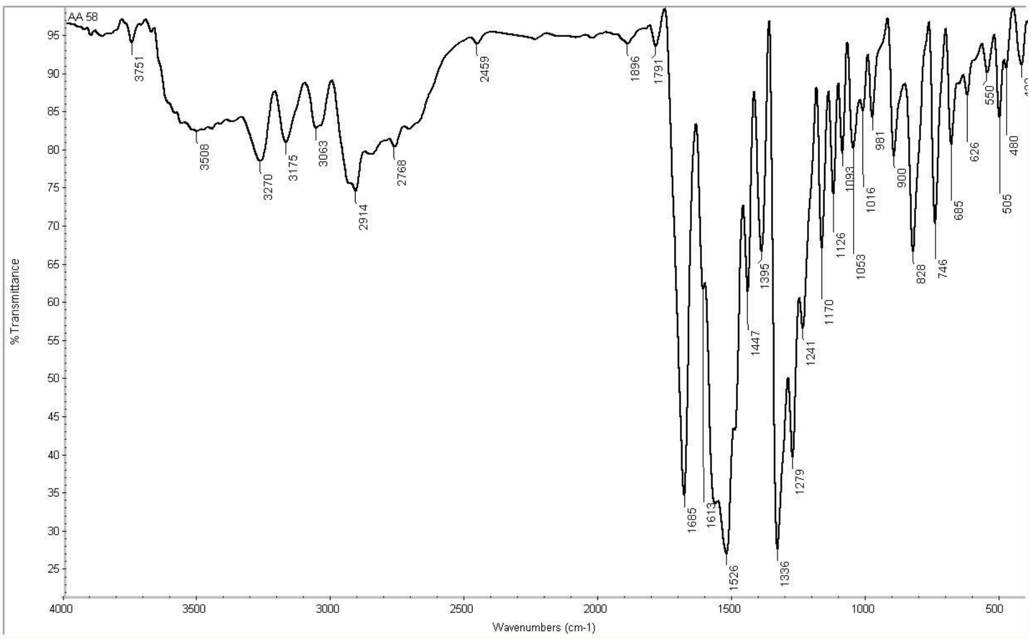
AA 58
% Transmittance
Wavenumbers (cm-1)
3751
3508
3270
3175
3063
2914
2768
2459
1896
1791
1685
1613
1526
1447
1395
1336
1279
1241
1170
1126
1093
1053
1016
981
900
828
746
685
626
550
505
480

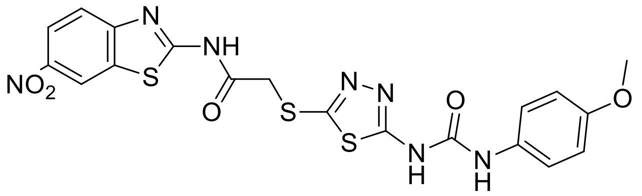
N
NH
NO_2
S
O
S
N
N
S
O
N
H
N
H
O

AA 59

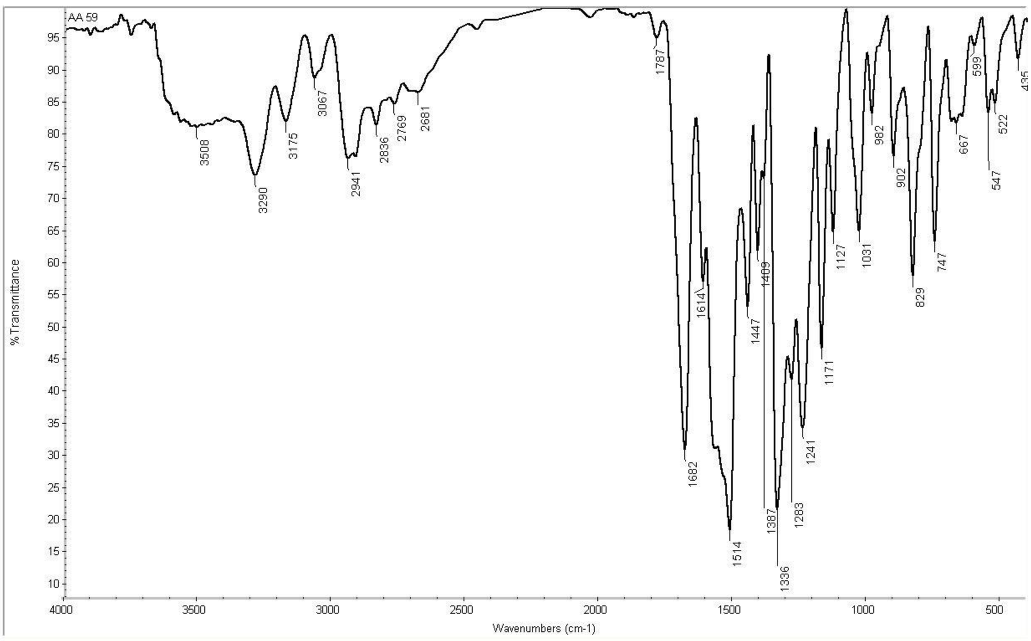
AA 59
% Transmittance
Wavenumbers (cm-1)
3508
3290
3175
3067
2941
2836
2769
2681
1787
1682
1614
1514
1447
1409
1387
1336
1283
1241
1171
1127
1031
982
902
829
747
667
599
547
522
435
